# A mitochondrial lipid metabolism–related gene signature predicts prognosis and immune landscape in colorectal cancer

**DOI:** 10.3389/fimmu.2025.1669678

**Published:** 2025-11-10

**Authors:** Hou Wang, Kai Zhang, Yueqiu Wang, Mengyun Chen, Mingchen Zhang

**Affiliations:** 1 Department of Endocrinology and Metabolism, Ningbo No.2 Hospital, Ningbo, Zhejiang, China; 2 Department of Endocrine and Metabolic Diseases, Shanghai Institute of Endocrine and Metabolic Diseases, Ruijin Hospital, Shanghai Jiao Tong University School of Medicine, Shanghai, China; 3 Department of Surgery, Shanghai Key Laboratory of Gastric Neoplasms, Shanghai Institute of Digestive Surgery, Ruijin Hospital, Shanghai Jiao Tong University School of Medicine, Shanghai, China

**Keywords:** colorectal cancer, prognostic biomarker, mitochondrial lipid metabolism, tumor microenvironment, immunotherapy, drug sensitivity

## Abstract

**Background:**

Colorectal cancer (CRC) is a highly aggressive gastrointestinal malignancy with significant global health consequences. While mitochondrial lipid metabolism genes are known to influence CRC progression, their prognostic relevance remains inadequately explored.

**Methods:**

This study systematically evaluated the expression profiles and prognostic significance of mitochondrial lipid metabolism-related genes in CRC patients. A risk model was constructed using data from the TCGA and GEO databases. Additionally, we examined the tumor microenvironment (TME), immune cell infiltration, tumor mutation burden, microsatellite instability (MSI), and drug sensitivity. Key genes associated with core mitochondrial lipid metabolism were identified and functionally validated through a series of *in vitro* cellular experiments.

**Results:**

Mitochondrial lipid metabolism-associated genes were identified, including ABHD4, ABHD8, HDHD5, PNPLA4, GK5, CPT2, YJEFN3, CRYAB, HSPA1A, MAPK1, ATG7, HDAC3, and ACAT2. A nomogram integrating the risk score with key clinical variables (pTNM stage and age) was developed to predict patient outcomes. Significant variations in immune cell infiltration were observed between risk groups. Immune microenvironment analysis revealed significant differences in immune cell infiltration between risk groups, and the risk score was significantly correlated with the expression of TME-related genes and immune checkpoint molecules, indicating a markedly immunosuppressive microenvironment in the high-risk group. Additionally, TIDE analysis showed that combining the risk score with immune, stromal scores and MSI could more effectively predict the benefit of immunotherapy. Furthermore, *in vitro* experiments demonstrated that knockdown of two key genes, ABHD4 and YJEFN3, significantly suppressed CRC cell proliferation, migration, and invasion, supporting their potential oncogenic roles.

**Conclusions:**

This mitochondrial lipid metabolism-based risk model represents a promising prognostic biomarker, offering potential guidance for personalized therapeutic strategies in CRC management.

## Introduction

Colorectal carcinoma (CRC) ranks as the third most prevalent malignancy globally, accounting for an estimated 1.9 million new cases annually (9.6% of total cancer incidence). It stands as the second most lethal oncologic disease, causing nearly 0.9 million deaths per year (9.3% of global cancer mortality) (1). Epidemiologic studies reveal a distinct geographic pattern: while CRC rates are stabilizing or declining in highly industrialized nations (albeit maintaining elevated absolute numbers), developing countries are experiencing rapid increases in both incidence and mortality (2). The clinical outlook is particularly grim for metastatic CRC cases, demonstrating dismal 5-year survival statistics under 20% (3). These concerning epidemiologic data underscore the urgent need for two critical advancements: (1) identification of more accurate prognostic indicators, and (2) discovery of novel molecular targets for therapeutic intervention in colorectal tumorigenesis.

Solid tumors evolve under nutrient-poor conditions, necessitating metabolic adaptations for survival (4). Clinical metabolomic data reveal that solid tumors frequently exhibit marked hypoglycemia. Remarkably, despite glucose deprivation, tumor tissues maintain relatively stable ATP levels, suggesting that alternative, glucose-independent energy production pathways may be activated in cancer cells (5). In particular, during metastatic progression, circulating tumor cells face severe metabolic stress due to impaired glucose uptake (6). From a metabolic perspective, fatty acid metabolism exhibits nutrient-dependent behavior: under nutrient-replete conditions, cells favor anabolic fatty acid synthesis (FAS), whereas nutrient deprivation triggers a shift toward catabolic fatty acid oxidation (FAO). This metabolic switch is tightly regulated by the acetyl-CoA carboxylase (ACC) enzyme family (7). Although recent studies underscore the importance of FAO in tumor metastasis, the upstream regulatory networks and precise mechanisms by which FAO contributes to malignant transformation remain to be fully elucidated. Recent research has demonstrated that the small-molecule compound CPI-613 exerts significant regulatory effects on mitochondrial lipid metabolism, as confirmed by multiple studies (8). A key target of CPI-613 is acetyl-CoA carboxylase (ACC), a central enzyme in lipid metabolism. Its inhibition induces metabolic reprogramming in tumor cells. Notably, in pancreatic cancer models, CPI-613 suppresses lipid metabolism through the activation of the AMPK signaling pathway, thereby exerting antitumor effects (9).

The tumor microenvironment is a specialized niche where tumor and host cells interact, and its metabolic characteristics profoundly influence anti-tumor immune responses and therapeutic outcomes. Recent studies have shown that tumor cells can evade immune surveillance by reprogramming the metabolic profiles of immune cells, while immune cells reciprocally modulate tumor behavior through metabolic feedback, forming a complex tumor-immune metabolic network (10). Solid tumors actively acquire lipid nutrients via specialized uptake mechanisms: tumor cells induce adjacent adipocytes to release free fatty acids (FFAs) and cholesterol, which are then internalized through fatty acid transport proteins (FATPs) (11). Within the TME, persistent oxidative stress arises from multiple sources: infiltrating neutrophils generate significant reactive oxygen species (ROS) during a respiratory burst, while enzymatic reactions mediated by lipoxygenase (LOX), cyclooxygenase (COX), and other oxidases further exacerbate oxidative pressure. Collectively, these processes establish a pronounced lipid peroxidation microenvironment, leading to the accumulation of cytotoxic oxidized lipid species such as oxidized low-density lipoprotein (ox-LDL) and malondialdehyde (MDA) (12). This lipid-rich milieu exerts dual regulatory effects on tumor immune responses: elevated fatty acid levels activate the PPARα signaling pathway in T cells, promoting FAO and oxidative phosphorylation (OXPHOS), whereas fatty acid depletion impairs T cell proliferation and antitumor activity (13). Thus, mitochondrial lipid metabolism is intricately linked to the immunosuppressive nature of the TME in CRC.

Lipid-targeting strategies demonstrate superior efficacy in cancer immunotherapy compared to glucose modulation, as tumors adapt more readily to glucose-targeted interventions. Importantly, glucose-targeting agents may inadvertently impair glucose-dependent antitumor immune cells, while protumor immune cells predominantly depend on lipid metabolism. Moreover, tumor cells themselves appear more vulnerable to disruptions in lipid homeostasis. Existing lipid-lowering drugs and NSAIDs can be repurposed to address lipid dysregulation within the tumor microenvironment (14). High-density lipoprotein (HDL) has been shown to downregulate the secretion of chemokines, such as CXCL16, via the SR-B1 receptor pathway (15). Interestingly, a reverse regulatory mechanism has been observed where certain chemokine family members, particularly CX3CL1, contribute to maintaining macrophage lipid homeostasis, although the precise signaling pathways involved remain to be fully elucidated (16).

Recognizing the crucial role of mitochondrial lipid metabolism in tumor development, identifying biomarkers related to mitochondrial lipid metabolism for colorectal cancer (CRC) prognosis represents a promising research direction. Although mitochondrial lipid metabolism is essential in cancer, the underlying biological mechanisms and therapeutic interventions based on mitochondrial lipid metabolism remain poorly defined. While numerous studies have developed prognostic models to predict CRC patient survival (17, 18), few have specifically focused on models linked to mitochondrial lipid metabolism that effectively predict prognosis and immunotherapy responsiveness in colorectal and rectal adenocarcinomas. In our study, we constructed a risk scoring model based on mitochondrial lipid metabolism, and further examined the relationship between risk scores and TME characteristics, including immune cell infiltration, immune checkpoint expression, and immunotherapy responses, while also assessing drug sensitivity across 198 compounds. Overall, our mitochondrial lipid metabolism-based risk model serves as a robust prognostic biomarker for CRC, offering valuable guidance for personalized treatment strategies. By linking mitochondrial lipid metabolism to an immunosuppressive TME, our model enhances our understanding of CRC pathogenesis and paves the way for improved therapeutic interventions.

## Materials and methods

### Data collection

The study analyzed genomic and clinical data from public repositories including RNA-sequencing profiles and microsatellite instability status for 620 colorectal adenocarcinoma specimens from The Cancer Genome Atlas (TCGA), along with matched clinical metadata from UCSC Xena. Validation of the 13-gene prognostic signature was conducted using the GEO dataset GSE39582, which comprises 585 colorectal cancer samples. To identify genes associated with lipid metabolism (LMRGs), the “Lipid Metabolism” category from the Molecular Signatures Database (MSigDB) was first examined. The candidate list was then refined by cross-referencing with the MitoCarta 3.0 database and relevant literature to extract genes with established roles in mitochondrial lipid metabolism. Expression profiles from colorectal cancer datasets were further analyzed to validate their biological relevance. In addition, bioinformatic analyses, including pathway enrichment, were applied to ensure that the final gene set accurately represented mitochondrial lipid metabolic processes. Through this multi-step screening strategy, a robust set of candidate genes was compiled for subsequent prognostic modeling in colorectal cancer.

### Construction and validation of prognostic mitochondrial lipid metabolism-related risk score signature

In this study, a comprehensive differential gene expression analysis was performed using the “limma” R package. Specifically, a threshold of |log2FC| > 1.3 and FDR < 0.05 was employed to identify differentially expressed genes. This threshold, adopted based on commonly applied criteria in previous CRC transcriptomic studies, was chosen to balance sensitivity and specificity (19). Comparisons were conducted between tumor and normal tissues as well as between high- and low-risk groups, and the results were visualized using volcano plots and Venn diagrams. Subsequently, we conducted univariate Cox regression to assess prognostic significance, followed by LASSO regression for feature selection, ultimately establishing a 13-gene risk signature calculated as:


$$\text{Risk score }=\;\sum \left({\text{β}}_{\text{i}}\;\times {\text{ ExpGene}}_{\text{i}}\right)$$

In this formula, expgene denotes the expression value of each gene, i represents the total count of signature genes (n=13), and β_i_ corresponds to the LASSO-derived regression coefficient for each gene. Using the median risk score as the cutoff threshold, patients were stratified into distinct high-risk and low-risk subgroups. Demographic and clinicopathological parameters (including sex, age at diagnosis, and TNM staging) were extracted from TCGA clinical records. Both univariate and multivariate Cox proportional hazards models were employed to assess the independent prognostic value of the risk signature, with statistical significance defined as p<0.05 (two-tailed). The predictive accuracy of this 13-gene classifier was externally validated in the GSE39582 cohort through multiple approaches: (1) time-dependent receiver operating characteristic (ROC) curve analysis, (2) risk stratification visualization, and (3) calculation of Harrell’s concordance index (C-index). All gene annotations were verified against the NCBI database.

### Construction and validation of nomogram

The study established a prognostic nomogram by integrating molecular risk scores with clinical parameters (age, TNM stage) through univariate and multivariate Cox regression analyses (P<0.05 significance threshold). The nomogram assigned weighted points to each predictor, with total scores enabling individualized 1-, 3-, and 5-year survival probability estimation. Model performance was validated using time-dependent ROC curves (discrimination), bootstrap-corrected calibration plots (accuracy), and decision curve analysis (clinical utility), demonstrating robust predictive capability for personalized outcome assessment.

### Gene ontology, Kyoto encyclopedia of genes and genomes analyses, and gene set enrichment analyses

Functional enrichment analysis was performed using R software (version 4.3.3) with the following packages: clusterProfiler for gene set enrichment, org.Hs.eg.db for gene annotation, enrichplot for visualization, and ggplot2 for graphical representation. We specifically examined differentially expressed genes (DEGs) associated with mitochondrial lipid metabolism and those distinguishing high- versus low-risk patient groups. Statistically significant functional terms were identified using a false discovery rate (FDR) threshold of <0.05 after multiple testing correction. Curated sets v7.4 collections from the MSigDB were used for GSEA, performed with GSEA 4.2.1 software. The total transcriptome of tumor samples was analyzed.

### Tumor microenvironment

Stromal scores and immune scores were calculated using the ESTIMATE algorithm in R (version 4.3.3) “estimate” package. The TME-related biomarker list was extracted from GSEA (http://www.gsea-msigdb.org/gsea/index.jsp). RNA-sequencing expression (level 3) profiles and clinical information for COADREAD were downloaded from the TCGA dataset (https://portal.gdc.cancer.gov/). To obtain robust immune score evaluations, we utilized the immunedeconv R package to implement the CIBERSORT algorithm.

### Prediction of therapeutic sensitivity in patients with different risk scores

This study systematically evaluated the predictive accuracy of our risk stratification model for both conventional and novel therapies by integrating multiple computational approaches. Using the “oncoPredict” R package (v1.2.0) in R (v4.3.3), we calculated the normalized half-maximal inhibitory concentrations (IC50) for 138 FDA-approved chemotherapeutic and targeted agents, referencing the Genomics of Drug Sensitivity in Cancer (GDSC) database (v8.2). Concurrently, immunotherapy response potential was assessed through the Tumor Immune Dysfunction and Exclusion (TIDE) algorithm, which evaluates immune evasion mechanisms and predicts checkpoint inhibitor responsiveness. This comprehensive analysis provides a robust framework for predicting therapeutic efficacy across diverse treatment modalities.

### Mutation analysis

Somatic mutation data for colorectal adenocarcinoma (COADREAD) were obtained from cBioPortal (https://www.cbioportal.org) and analyzed using the “maftools” R package (v3.5.1) to visualize mutation profiles and calculate tumor mutational burden (TMB). Microsatellite instability (MSI) status was retrieved from the TCGA dataset via the Genomic Data Commons (GDC) portal. All analyses followed standardized bioinformatics workflows with quality control, allowing comprehensive assessment of mutation patterns and clinically relevant MSI features across risk groups.

### Cell lines and cell culture

The human colorectal cancer (CRC) cell lines RKO and HCT116 were obtained from the American Type Culture Collection (ATCC, USA). Cell line authentication was performed using short tandem repeat (STR) profiling, and all cell lines were confirmed to be free of mycoplasma contamination. Cells were cultured in Dulbecco’s Modified Eagle Medium (DMEM; Meilunbio, Dalian, China) supplemented with 10% fetal bovine serum (FBS; Gibco, Grand Island, NY, USA), 100 U/mL penicillin, and 100 µg/mL streptomycin. Cultures were maintained in a humidified incubator at 37°C with 5% CO_2_.

### Lentiviral-mediated knockdown of ABHD4 and YJEFN3

To achieve stable knockdown of ABHD4 and YJEFN3, target shRNA sequences were cloned into the pGreenPuro (CMV) vector. The shRNA sequence targeting ABHD4 was 5′-CCGGACTTCAAACGCAAGTTT-3′, and that for YJEFN3 was 5′-AGAGCGGAGCTTAGCTCAAAT-3′. Lentiviral particles were produced and used to transduce RKO and HCT116 cells. Cells were seeded in 6-well plates and infected when they reached 60–80% confluence using viral supernatant supplemented with 10 μg/mL polybrene (Sigma-Aldrich, USA) overnight. Following infection, the medium was replaced, and 48 h later, puromycin (10 μg/mL; Sigma-Aldrich, USA) was added to select for stably transduced cells, resulting in the establishment of the RKO/shABHD4, HCT116/shABHD4, RKO/shYJEFN3, and HCT116/shYJEFN3 cell lines.

### Western blotting

Cells were washed with cold PBS and lysed in RIPA buffer (Kangwei, Beijing, China) supplemented with phosphatase inhibitors (Roche, Switzerland). Protein concentrations were determined using the BCA assay (Pierce, USA). Equal amounts of protein (20 μg) were separated by 10% SDS-PAGE and transferred to 0.22 μm PVDF membranes (Millipore, USA). Membranes were blocked with 5% non-fat milk in TBST and incubated overnight at 4°C with primary antibodies: anti-GAPDH (Proteintech, 0.02 µg/mL), anti-ABHD4 (Thermo Fisher), and anti-YJEFN3 (Atlas Antibodies). After washing, membranes were incubated with HRP-conjugated secondary antibody (Thermo Fisher) and developed using ECL substrate. Signals were detected using the Tanon 5200 system (Tanon, China).

### CCK-8 assay

Cell proliferation was assessed using the Cell Counting Kit-8 (CCK-8; Dojindo, Kumamoto, Japan) according to the manufacturer’s instructions. Briefly, CRC cells were seeded into 96-well plates at a density of 1,000 cells/well in 200 μL of complete medium. After incubation with 20 μL of CCK-8 solution for 2 h, absorbance at 450 nm (OD450) was measured using a microplate reader (BioTek, VT, USA). All experiments were performed in triplicate.

### Migration and invasion assays

For migration assays, 1 × 10^5^ cells suspended in serum-free medium were seeded in the upper chamber of 24-well Transwell inserts (8 μm pore size; Corning, MA, USA). Medium containing 10% FBS was added to the lower chamber as a chemoattractant. After 12 h of incubation, migrated cells on the lower surface of the membrane were fixed and stained with 0.1% crystal violet for 30 min.

For invasion assays, inserts were pre-coated with diluted Matrigel (BD Biosciences, San Jose, CA, USA). Cells were seeded in the same manner, and after 24 h, invaded cells were fixed and stained similarly. Stained cells were imaged and counted under a microscope in ten randomly selected fields. The average number of migrated or invaded cells was calculated.

### Wound-healing assay

For wound-healing assays, 4 × 10^5^ cells/well were seeded in 24-well plates and allowed to form a monolayer. A scratch was made using a sterile pipette tip, and images were captured at 0 h and 24 h. The migration rate was analyzed using ImageJ software (NIH, USA). Experiments were performed in triplicate.

### Statistical analysis

All statistical analyses were performed using R software (version 4.3.3) and GraphPad Prism (version 10.0.1), employing Student’s t-tests for continuous variables (risk scores, stromal/immune scores, tumor purity, and TMB), χ² tests for categorical variables (immunotherapy response and clinical factors), Spearman correlation for association analyses, and the concordance index (C-index) to evaluate the predictive power of age and risk scores for overall survival (OS), along with univariate and multivariate Cox regression analyses to assess the prognostic significance of mitochondrial lipid metabolism-related genes and clinical characteristics, with a two-tailed P-value < 0.05 considered statistically significant. For GO and KEGG enrichment analyses, the Benjamini-Hochberg procedure was applied to adjust for multiple comparisons, and results were reported as FDR-adjusted p-values. For Gene Set Enrichment Analysis (GSEA), we used normalized enrichment scores (NES) and FDR q-values to assess significance, following standard GSEA criteria, with FDR < 0.25 considered statistically significant. For immune cell infiltration comparisons, FDR correction was also applied when evaluating differences in immune cell populations between the high- and low-risk groups. For genome-wide survival screening, p-values from univariate Cox regression analyses were adjusted using the Benjamini-Hochberg method to control the false discovery rate (FDR). For survival analysis of selected candidate genes or model components, raw p-values were reported without multiple testing correction.

## Results

### Identification of DEGs related to mitochondria lipid metabolism and functional enrichment analysis in COADREAD

**Figure 1:**
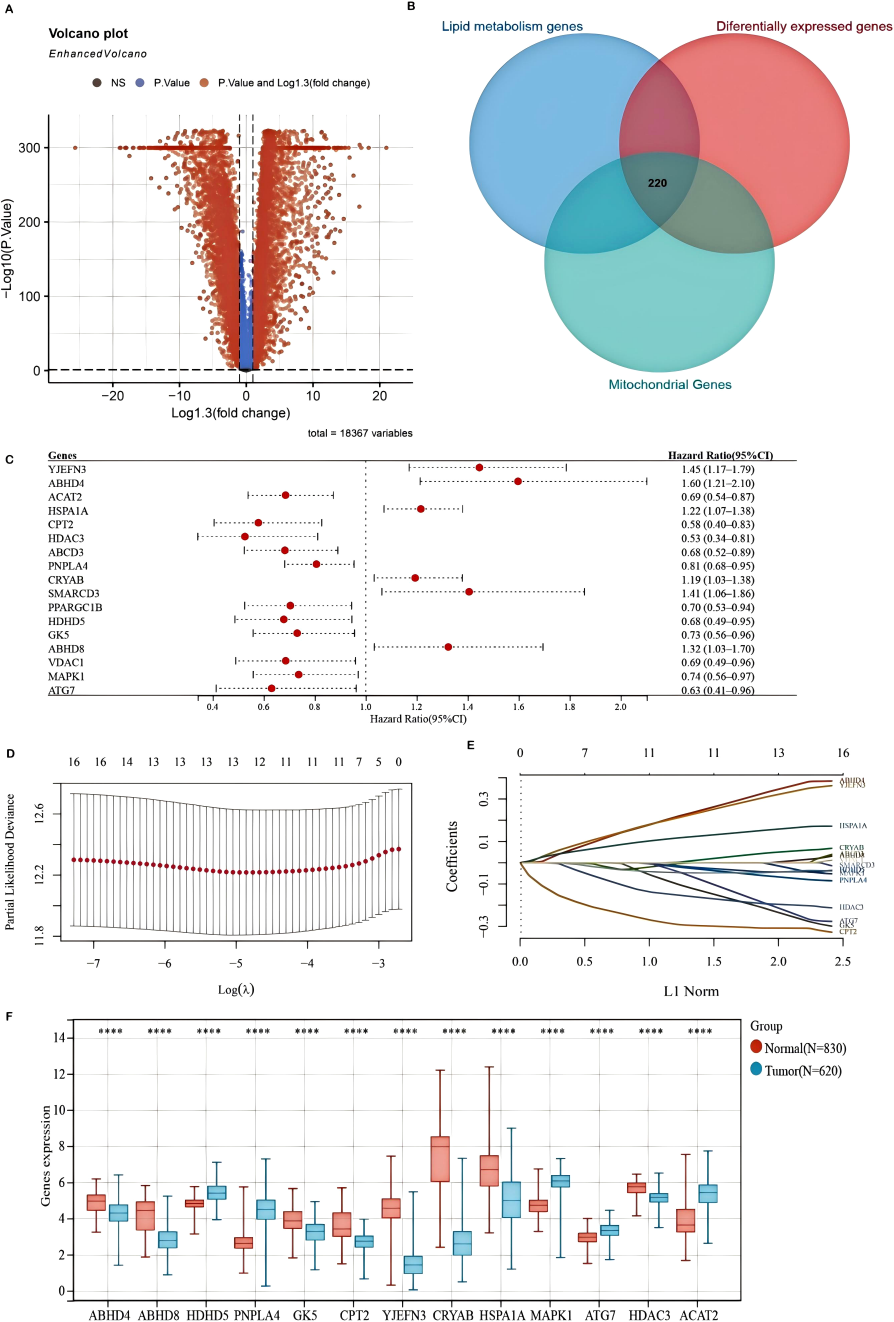
Differentially expressed genes (DEGs) associated with mitochondrial lipid metabolism and the construction of a prognostic model in the TCGA-COADREAD cohort **(A)** A volcano plot illustrating the expression differences between COADREAD tumor and normal tissues, identifying 10,852 genes. **(B)** A Venn diagram depicting the intersection among DEGs, lipid metabolism-related genes, and mitochondrial genes, resulting in 220 hub genes. **(C)** A forest plot evaluating 17 genes related to prognosis, demonstrating their impact on patient outcomes. **(D, E)** LASSO regression analyses of the 17 overall survival (OS)-related genes, including cross-validation to determine the optimal tuning parameter (log[λ] on the x-axis and partial likelihood deviance on the y-axis, with red dots indicating deviations ± standard error). **(F)** Expression levels of the 13 prognostically significant core genes in the TCGA-COADREAD cohort. Significance levels are indicated as ****P < 0.0001.

A comprehensive analysis was conducted to identify differentially expressed genes (DEGs) associated with mitochondrial lipid metabolism in colorectal and rectal adenocarcinoma (COADREAD). The overall study design is illustrated in **Supplementary Figure S1**. A total of 10,852 DEGs were detected, comprising 5,839 significantly downregulated and 5,013 significantly upregulated genes, which were visualized using volcano plots to compare tumor and normal samples (**Figure 1A**). Mitochondrial lipid metabolism gene set was selected from the MSigDB database. This gene set was curated through literature mining and experimental validation and comprises genes involved in mitochondrial lipid metabolism pathways. These genes are extensively associated with lipid synthesis (anabolism), degradation (catabolism), and regulation, and are known to play critical roles in energy homeostasis, membrane integrity, and cell signaling. To further refine the selection, we identified 220 mitochondrial lipid metabolism-related genes by integrating the results of Gene Set Enrichment Analysis (GSEA) with the DEG dataset (**Figure 1B**).

Gene Ontology (GO) enrichment analysis demonstrated that these DEGs were primarily involved in biological processes such as lipid metabolism and small molecule metabolic processes (**Supplementary Figure S2A**). In terms of cellular components, the identified genes were predominantly enriched in the mitochondrial membrane and mitochondrial envelope (**Supplementary Figure S2B**). Functionally, they exhibited catalytic activity and anion binding (**Supplementary Figure S2C**).

Furthermore, KEGG pathway analysis highlighted key pathways associated with these DEGs, including insulin resistance, glycerophospholipid metabolism, and fatty acid metabolism (**Supplementary Figure S2D**). Notably, both insulin resistance and glycerophospholipid metabolism have been linked to colorectal cancer (CRC) progression, potentially facilitating tumor growth and survival through the PI3K-AKT and mTOR signaling pathways. Collectively, these findings enhance our understanding of the molecular mechanisms through which mitochondrial lipid metabolism-related DEGs influence COADREAD development and progression.

### Construction and validation of a mitochondrial lipid metabolism-related risk signature

To develop a mitochondrial lipid metabolism-related risk signature, we initially identified 17 prognostic candidates for COADREAD from 220 differentially expressed mitochondrial lipid metabolism-related genes using univariate Cox regression analysis (P < 0.05). Assessment of mitochondrial lipid metabolism-related genes in predicting prognosis of CRC exhibited by forest plot are shown in **Figure 1C**. LASSO regression analysis further refined this list to 13 key genes (**Figures 1D, E**). These genes formed the basis of our prognostic model, detailed in **Supplementary Figure S3**. The risk score for each patient was determined using the following formula:


$$\begin{array}{c} \text{Risk score }=\;\left(0.3381\right)\;\times \text{ ABHD}4\;+\;\left(7\text{e}-04\right)\;\times \text{ ABHD}8\\ \;+\;\left(-0.0089\right)\;\times \text{ HDHD}5\;+\;\left(-0.0641\right)\\ \;\times \text{ PNPLA}4\;+\;\left(-0.2255\right)\;\times \text{ GK}5\;+\;\left(-0.3078\right)\\ \;\times \text{ CPT}2\;+\;\left(0.315\right)\;\times \text{ YJEFN}3\;+\;\left(0.0476\right)\\ \;\times \text{ CRYAB }+\;\left(0.1557\right)\;\times \text{ HSPA}1\text{A }+\;\left(-0.0437\right)\\ \;\times \text{ MAPK}1\;+\;\left(-0.2021\right)\;\times \text{ ATG}7\;+\;\left(-0.1944\right)\\ \;\times \text{ HDAC}3\;+\;\left(-0.0454\right)\;\times \text{ ACAT}2. \end{array}$$

Analysis of the TCGA-COADREAD dataset confirmed distinct expression patterns of mitochondrial lipid metabolism-related genes. Specifically, HDHD5, PNPLA4, MAPK1, ATG7, and ACAT2 were significantly upregulated, whereas ABHD4, ABHD8, GK5, CPT2, YJEFN3, CRYAB, HSPA1A, and HDAC3 were notably downregulated in tumor tissues compared to normal samples (**Figure 1F**). These alterations suggest a crucial role for these genes in shaping the tumor immune microenvironment and influencing colorectal cancer progression.

**Figure 2:**
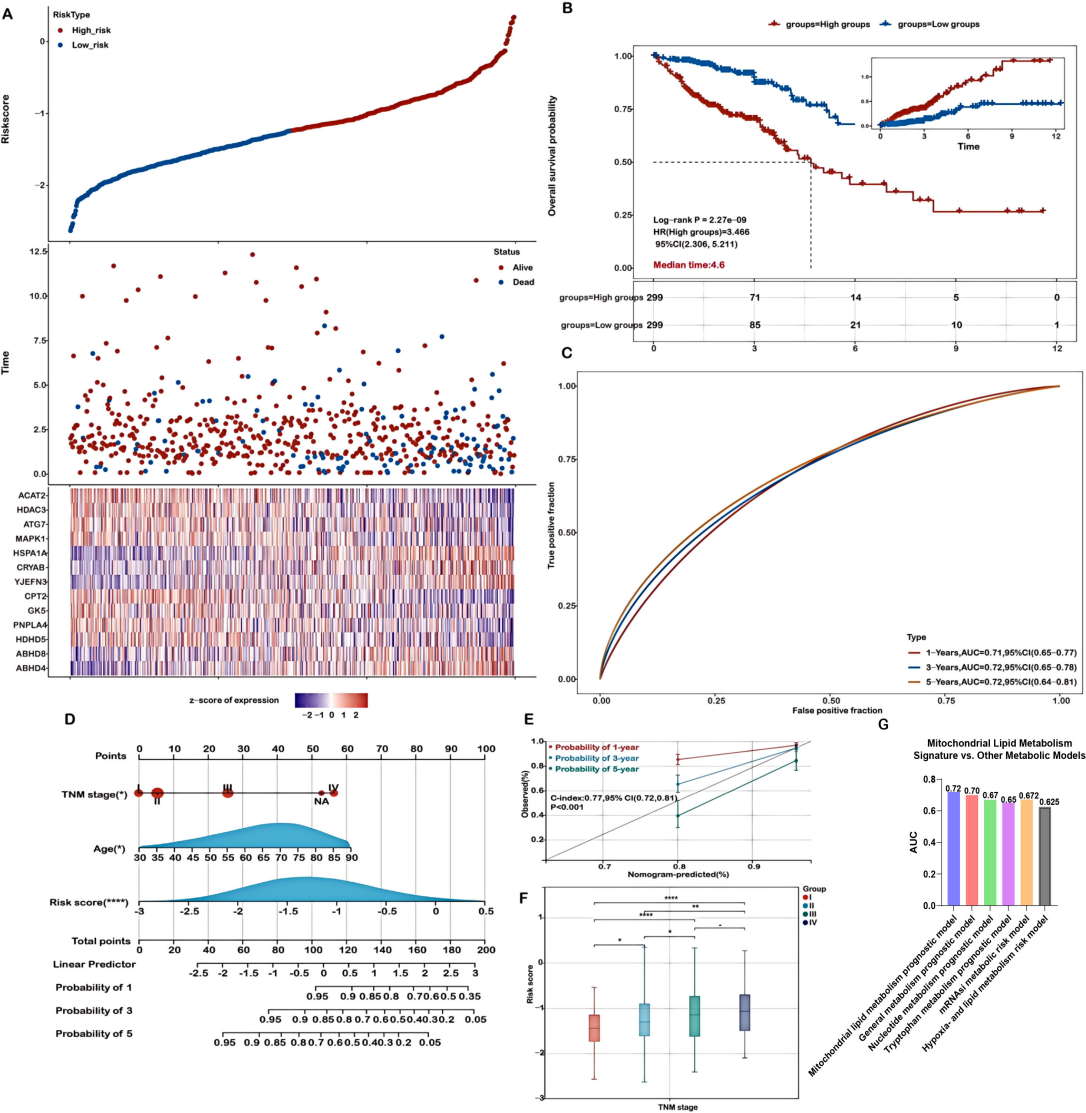
Evaluation of the prognostic model in the training cohort. **(A)** Distribution plots showing risk scores, survival status (blue indicates deceased, red indicates alive), and expression of the 13 model genes in the TCGA-COADREAD training set. **(B)** Kaplan-Meier survival curves comparing overall survival between high- and low-risk groups. **(C)** Receiver Operating Characteristic (ROC) curves for predicting 1-, 3-, and 5-year overall survival. **(D)** A nomogram integrating risk score with relevant clinical features. **(E)** Calibration curves demonstrating the concordance between predicted and actual survival probabilities at 1, 3, and 5 years. **(F)** Analysis of the association between risk scores and TNM stage in COADREAD patients. **(G)** Time-dependent ROC analysis comparing the 3-year overall survival predictive performance of the mitochondrial lipid metabolism signature with previously published metabolism-related models. Significance levels are indicated as ****P < 0.0001, **P < 0.01, *P < 0.05.

The association between risk scores and survival time, survival status, risk stratification, and gene expression profiles is illustrated in **Figure 2A**. Patients were classified into high- and low-risk groups based on the median risk score. Kaplan-Meier survival analysis indicated a significantly poorer overall survival (OS) for patients in the high-risk group (P = 2.27e-09, **Figure 2B**). The predictive capability of the prognostic model for 1-, 3-, and 5-year OS was assessed using ROC curves, yielding AUC values of 0.71, 0.72, and 0.72, respectively (**Figure 2C**), demonstrating its effectiveness in prognostic evaluation. In addition, we performed direct ROC comparisons between our 13-gene signature and several previously published metabolism-related prognostic models in CRC (**Figure 2G**). Notably, our model achieved the highest AUC value (0.72), surpassing those based on nucleotide metabolism (AUC = 0.67), general metabolism-related genes (AUC = 0.70), tryptophan metabolism–associated signatures (AUC = 0.65), mRNAsi-related metabolic risk scores (AUC = 0.672), and hypoxia- and lipid metabolism–related genes (AUC = 0.625). These findings indicate that our signature exhibits superior prognostic discriminatory power compared with existing models.

To further evaluate the model’s performance, validation was conducted using the GSE39582 dataset. Consistent with the TCGA-COADREAD training cohort, higher risk scores were associated with worse survival outcomes (**Supplementary Figures S4A, B**). The expression profiles of 13 genes in the validation dataset are displayed as heatmaps (**Supplementary Figure S4A**), and Kaplan-Meier analysis confirmed significantly poorer survival in high-risk patients (**Supplementary Figure S4B**). ROC curve analysis for 1-, 3-, and 5-year survival yielded AUC values of 0.68, 0.66, and 0.65, respectively (**Supplementary Figure S4C**), further validating the model’s robustness and clinical relevance.

### Construction and assessment of a prognostic model based on mitochondrial lipid metabolism-related genes

A nomogram was constructed to predict patient prognosis quantitatively by combining the risk score and essential clinical variables, supporting clinical decision-making. Both univariate and multivariate analyses revealed that the risk score, pTNM stage, and age were significant, marking them as independent prognostic factors (**Figure 2D**). The nomogram, which integrates these independent prognostic factors—risk score, pTNM stage, and age—was developed to forecast patient outcomes (**Figure 2D**). The calibration curves demonstrated a high level of agreement between the predicted and observed survival probabilities at 1-, 3-, and 5-year intervals (**Figure 2E**). Calibration curves were also generated to further validate the predictive reliability of the nomogram. The nomogram exhibited strong prognostic accuracy, reflected in a concordance index of 0.77 (95% CI: 0.72-0.81; p<0.001), confirming its predictive value.

Additionally, we evaluated the potential of risk scores derived from mitochondrial lipid metabolism-related genes as valuable biomarkers for patient stratification and prognosis in colorectal cancer. Our results revealed significant associations between these risk scores and established clinical stage parameters. Notably, higher risk scores were closely linked to advanced disease stages (Stage II-IV vs. I, and Stage IV vs. II, III; **Figure 2F**, P < 0.05), further supporting the utility of the risk score as a prognostic biomarker, particularly in later-stage colorectal cancer. Moreover, high risk scores were significantly associated with advanced T staging, lymph node metastasis (N staging), and distant metastasis (M staging), indicating that abnormal mitochondrial lipid metabolism may promote tumor invasion into deeper tissues and metastasis (**Supplementary Figure S5**).

### Functional enrichment analysis of DEGs in high-risk and low-risk groups

**Figure 3:**
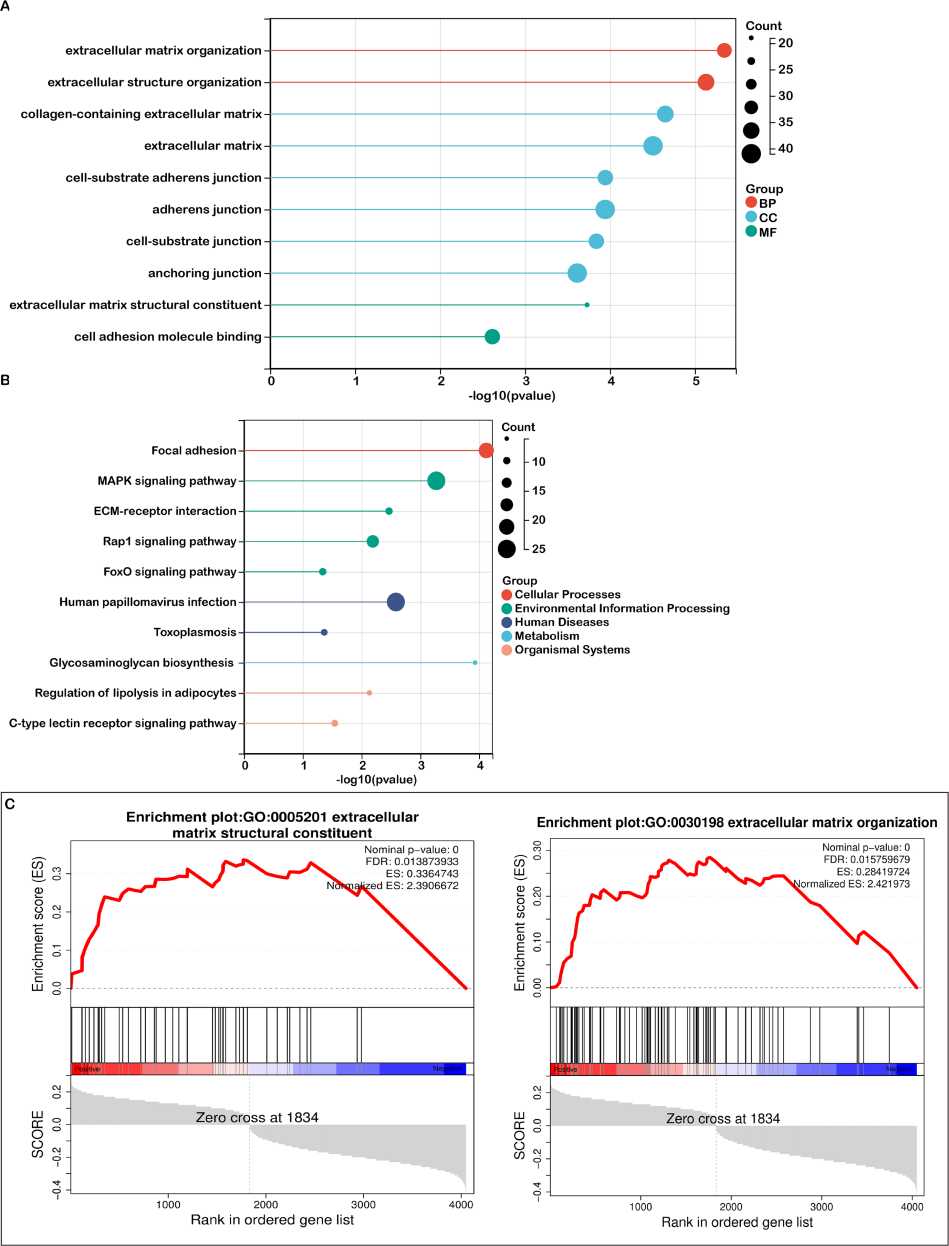
Pathway enrichment analysis in high- and low-risk groups. **(A)** A bubble plot presenting the top 10 significant Gene Ontology (GO) terms, color-coded by biological process (BP), cellular component (CC), and molecular function (MF), with associated genes listed. **(B)** A bubble plot depicting the top 10 significant KEGG pathways, with color distinctions representing each pathway and the related gene lists provided. **(C)** Gene Set Enrichment Analysis (GSEA) identifying distinct gene sets enriched in the high-risk group.

**Figure 4:**
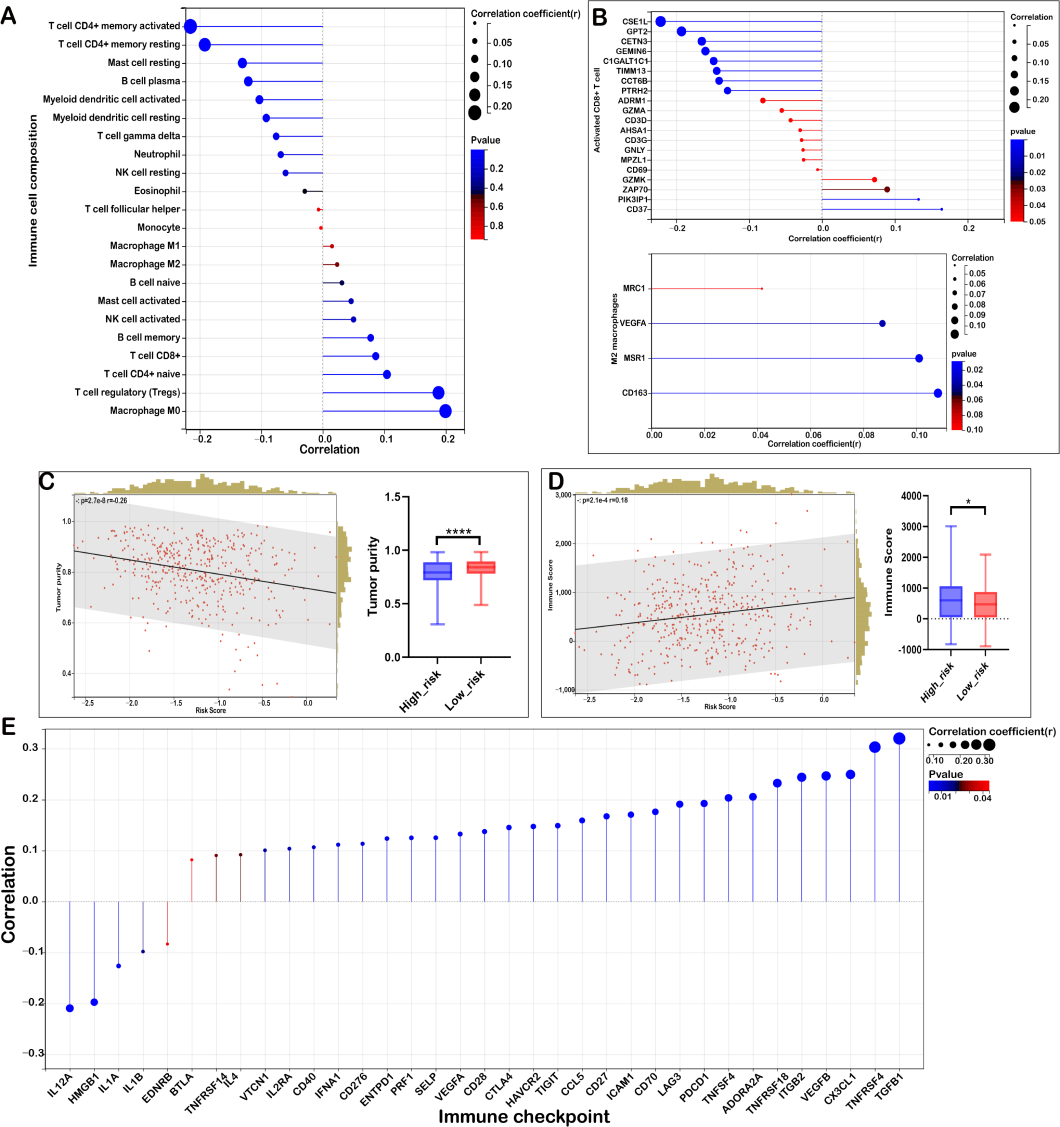
Association between risk score and immune microenvironment characteristics. **(A)** CIBERSORT analysis demonstrating the correlation between the risk score and various immune cell types. **(B)** Correlation between the risk score and the expression of signatures for activated CD8+ T cells and M2 macrophages. **(C)** Analysis of the relationship between the risk score and tumor purity, with distributions shown for each risk group. **(D)** Correlation between the risk score and immune score, along with corresponding group distributions. **(E)** Association between the risk score and the expression of immune checkpoint molecules. Significance levels: ****P < 0.0001, *P < 0.05

Functional enrichment analyses were performed on differentially expressed genes (DEGs) in both high-risk and low-risk groups. Gene Ontology (GO) enrichment analysis revealed that DEGs associated with biological processes were predominantly involved in extracellular matrix organization and extracellular structure organization (**Figure 4A**). For cellular components, DEGs were mainly related to cell-substrate adherens junctions, and collagen-containing extracellular matrix (**Figure 3A**). Regarding molecular functions, the DEGs were enriched in cell adhesion molecule binding and extracellular matrix structural constituents (**Figure 3A**).

KEGG pathway analysis identified the top 10 enriched pathways, including focal adhesion, MAPK signaling, ECM-receptor interactions, Rap1 signaling, human papillomavirus infection, FoxO signaling, toxoplasmosis, glycosaminoglycan biosynthesis, regulation of lipolysis in adipocytes, and C-type lectin receptor signaling (**Figure 3B**). Additionally, Gene Set Enrichment Analysis (GSEA) highlighted that mitochondrial lipid metabolism-related risk scores in the high-risk group were strongly associated with extracellular matrix structural constituent and extracellular matrix organization (**Figure 3C**).

Both the GO analysis and GSEA emphasized the extracellular matrix’s pivotal role in tumor biology, with terms such as extracellular matrix structural constituent and organization being prominent. KEGG analysis further revealed key pathways like focal adhesion and ECM-receptor interactions, both of which are critically involved in the TME. The interactions between cells and the ECM are fundamental to TME signaling, affecting cell adhesion, migration, and invasion. Since the TME is largely composed of extracellular matrix components and regulatory signaling molecules, our analyses point to several key pathways—ECM organization, receptor interactions, and collagen-related processes—as crucial players within the TME. These findings support the conclusion that TME-associated pathways are significantly enriched.

### Mitochondrial lipid metabolism-related risk score and TME signatures in COADREAD

**Figure 5:**
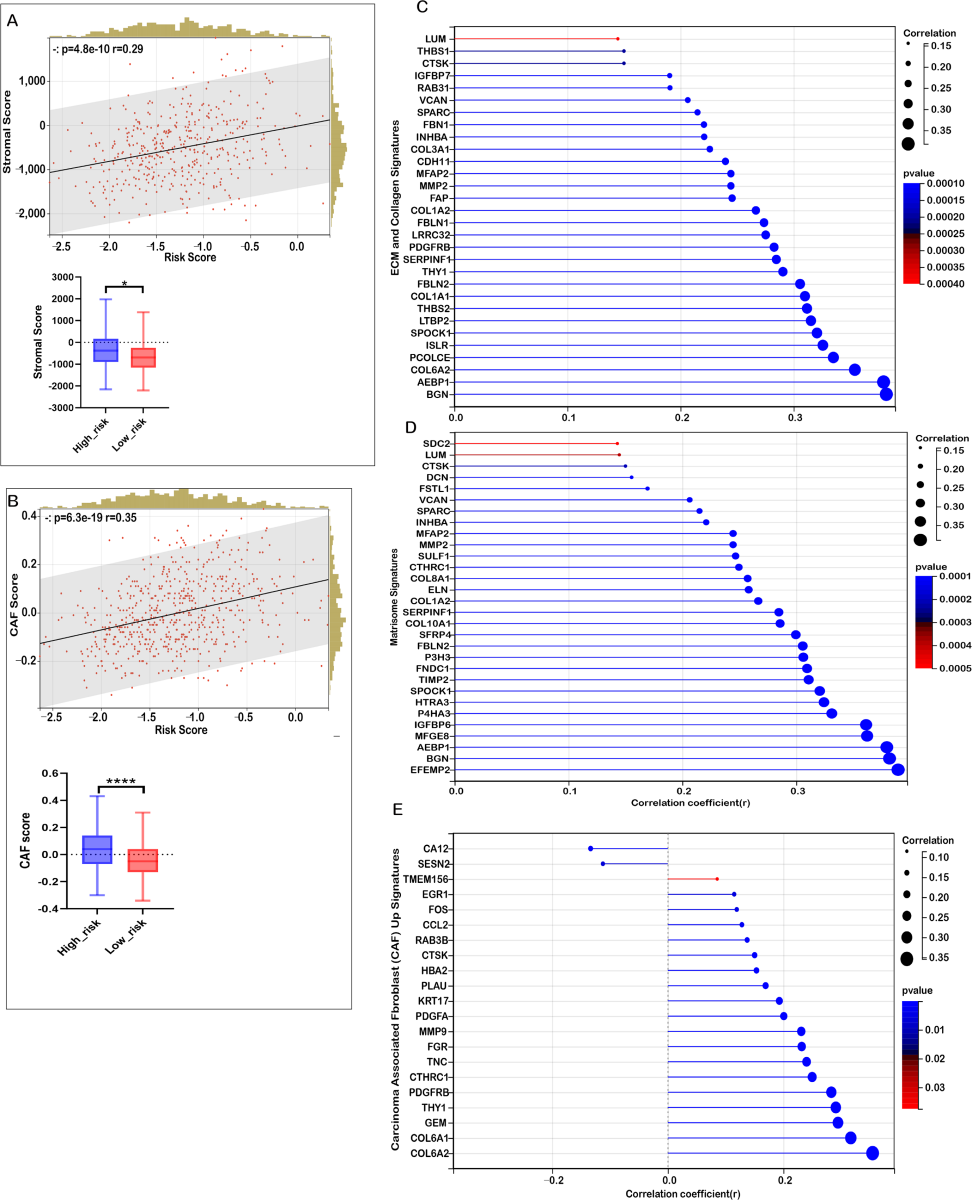
Correlation between risk score and tumor microenvironment (TME) signatures in COADREAD. **(A)** Analysis showing the association between stromal score and risk score, with distributions across low- and high-risk groups. **(B)** Examination of the relationship between carcinoma-associated fibroblast (CAF) score and risk score, including group distribution. **(C)** Correlation analysis of the risk score with extracellular matrix (ECM) and collagen gene signatures. **(D)** Correlation of the risk score with matrisome gene expression. **(E)** Association between the risk score and both upregulated and downregulated CAF signatures. Significance is indicated as ****P < 0.0001, *P < 0.05.

Based on the functional enrichment of TME-associated signaling pathways, we examined the relationship between the risk score and TME-related signatures. As depicted in **Figure 5A**, a strong positive correlation was observed between the risk score and stromal score in COADREAD, with the high-risk group showing higher stromal scores than the low-risk group. Additionally, we identified a significant positive correlation between the risk score and the cancer-associated fibroblast (CAF) score (**Figure 5B**), with the high-risk group exhibiting notably elevated CAF scores, highlighting the involvement of CAFs in CRC progression and prognosis.

Moreover, significant positive correlations were found between the risk score and the expression of various ECM-collagen signatures (**Figure 5C**), as well as matrisome and CAF signatures (**Figures 5D, E**). Our results suggest that mitochondrial lipid metabolism-related risk scores may influence matrix remodeling by regulating ECM-collagen and matrisome gene expression, affecting tumor microenvironment formation and function. In summary, we found a significant positive correlation between mitochondrial lipid metabolism risk scores and ECM components, CAF activity, and ECM-related gene expression in the tumor microenvironment. This indicates that mitochondrial lipid metabolic abnormalities impact tumor cell metabolism and indirectly promote tumor cell proliferation, invasion, and metastasis by regulating ECM and CAF activity in the microenvironment.

### Mitochondrial lipid metabolism-associated risk score reveals the characteristics and heterogeneity of the immunosuppressive microenvironment in high-risk patients

The tumor immune microenvironment (TIME) plays a critical role in determining therapeutic efficacy and prognosis in malignant tumors. Understanding the relationship between risk scores and immune cell infiltration in COADREAD is essential for improving treatment strategies. we applied the CIBERSORT algorithm to estimate immune cell proportions. The results revealed distinct correlations between risk score and various immune cell subtypes.

As shown in **Figure 4A**, regulatory T cells (Tregs) were significantly positively correlated with the risk score (r = 0.188, P < 0.05), suggesting the presence of a potentially immunosuppressive microenvironment in high-risk patients. Similarly, M0 macrophages (r = 0.199, P < 0.05) exhibited a strong positive correlation, indicating a prevalence of undifferentiated macrophages that may differentiate into either pro-inflammatory (M1) or immunosuppressive (M2) subtypes.

Furthermore, naïve B cells (r = 0.031), memory B cells (r = 0.078), CD8+ T cells (r = 0.086), and naïve CD4+ T cells (r = 0.104) showed weak positive correlations with the risk score. Notably, activated mast cells (r = 0.046) and activated NK cells (r = 0.050) also demonstrated weak positive correlations.

Several immune cell subtypes were negatively correlated with the risk score. Resting memory CD4+ T cells (r = -0.192, P < 0.05) and activated memory CD4+ T cells (r = -0.215, P < 0.05) exhibited strong negative correlations, suggesting that a reduced population of memory T cells may contribute to immune dysfunction in high-risk patients. Additionally, plasma B cells (r = -0.121), resting mast cells (r = -0.131), and gamma delta T cells (r = -0.076) were negatively associated with the risk score. Other immune cells with negative correlations included resting NK cells (r = -0.060), resting dendritic cells (r = -0.092), activated dendritic cells (r = -0.103), eosinophils (r = -0.029), and neutrophils (r = -0.068), indicating potential suppression of innate immune responses in high-risk patients.

Furthermore, the risk score exhibited a negative correlation with activated CD8+ T cell signatures (**Figure 4B**), indicating a weakened anti-tumor immune response in high-risk individuals. Conversely, a positive correlation was observed between the risk score and activated M2 macrophage signatures (**Figure 4B**), highlighting the presence of an immunosuppressive environment in these patients.

The immune cell characteristics between the low-risk and high-risk groups (**Supplementary Figure S6**), are highly consistent with the results above. High-risk patients exhibited an immunosuppressive tumor microenvironment, with significant positive correlations of regulatory T cells and M0 macrophages with the risk score, suggesting enhanced immune evasion. Weak positive correlations were also observed for certain B cells, CD8+ T cells, and activated mast and NK cells. In contrast, memory CD4+ T cells, plasma B cells, dendritic cells, and other immune subtypes showed negative correlations, indicating a weakened immune response in high-risk patients. These findings suggest distinct immune landscapes that may influence immunotherapy outcomes.

To further evaluate the tumor immune microenvironment, we applied the ESTIMATE algorithm. A robust positive association was observed between the risk score and the immune score and high-risk group demonstrated an elevated immune score, suggesting partial immune infiltration (**Figure 4D**). Additionally, they showed a significantly higher stromal score (**Figure 5A**) and reduced tumor purity (**Figure 4C**). A strong positive correlation was identified between the risk score and both matrisome and cancer-associated fibroblast (CAF) signatures, while an inverse correlation was noted with activated CD8+ T cell signatures. These findings indicate that an enriched extracellular matrix and fibroblast-dominant microenvironment may suppress CD8+ T cell activation, fostering immune evasion in high-risk COADREAD patients. The close relationship between risk score, immune infiltration, and stromal components underscores the potential of targeting the immunosuppressive microenvironment as a therapeutic avenue, necessitating further research.

### Mitochondrial lipid metabolism-related risk score was associated immune checkpoint inhibitors and immunotherapy responses in COADREAD

Considering the potential of immune checkpoint inhibitors (ICIs) as a treatment for cancer, we examined the relationship between immune checkpoints and risk stratification. Our findings revealed that 36 immune checkpoints were significantly altered in the high-risk group (**Figure 4E**). Notably, the risk score showed a negative correlation (r > -0.1) with IL1A, IL1B, HMGB1, and IL12A, which are key mediators of inflammation and immune activation. This suggests a potential suppression of pro-inflammatory signaling pathways, leading to reduced antigen presentation and impaired CD8+ T cell activation in high-risk patients.

In contrast, the risk score was positively correlated (r > 0.1) with multiple immune checkpoints associated with both immune activation and suppression (**Figure 4E**). Upregulation of CD40, CD28, CD27, and TNFRSF4 suggests enhanced T cell co-stimulation; however, the simultaneous increase in inhibitory receptors such as PDCD1 (PD-1), CTLA4, LAG3, and TIGIT indicates a state of T cell exhaustion, which may contribute to immune evasion. Additionally, positive correlations with TGFB1 and ADORA2A highlight a highly immunosuppressive tumor microenvironment that could further inhibit effective anti-tumor responses.

The risk score was positively correlated (r > 0.1) with VEGFA, VEGFB, and ICAM1, suggesting enhanced angiogenesis that may restrict immune infiltration and promote tumor progression. Although PRF1 and CX3CL1 showed some cytotoxic potential, the overall immune landscape in high-risk patients is immunosuppressive. These results indicate that targeting immune checkpoints, angiogenesis, and immunosuppressive pathways (e.g., TGF-β, adenosine signaling) may improve anti-tumor immunity in high-risk patients.

**Figure 6:**
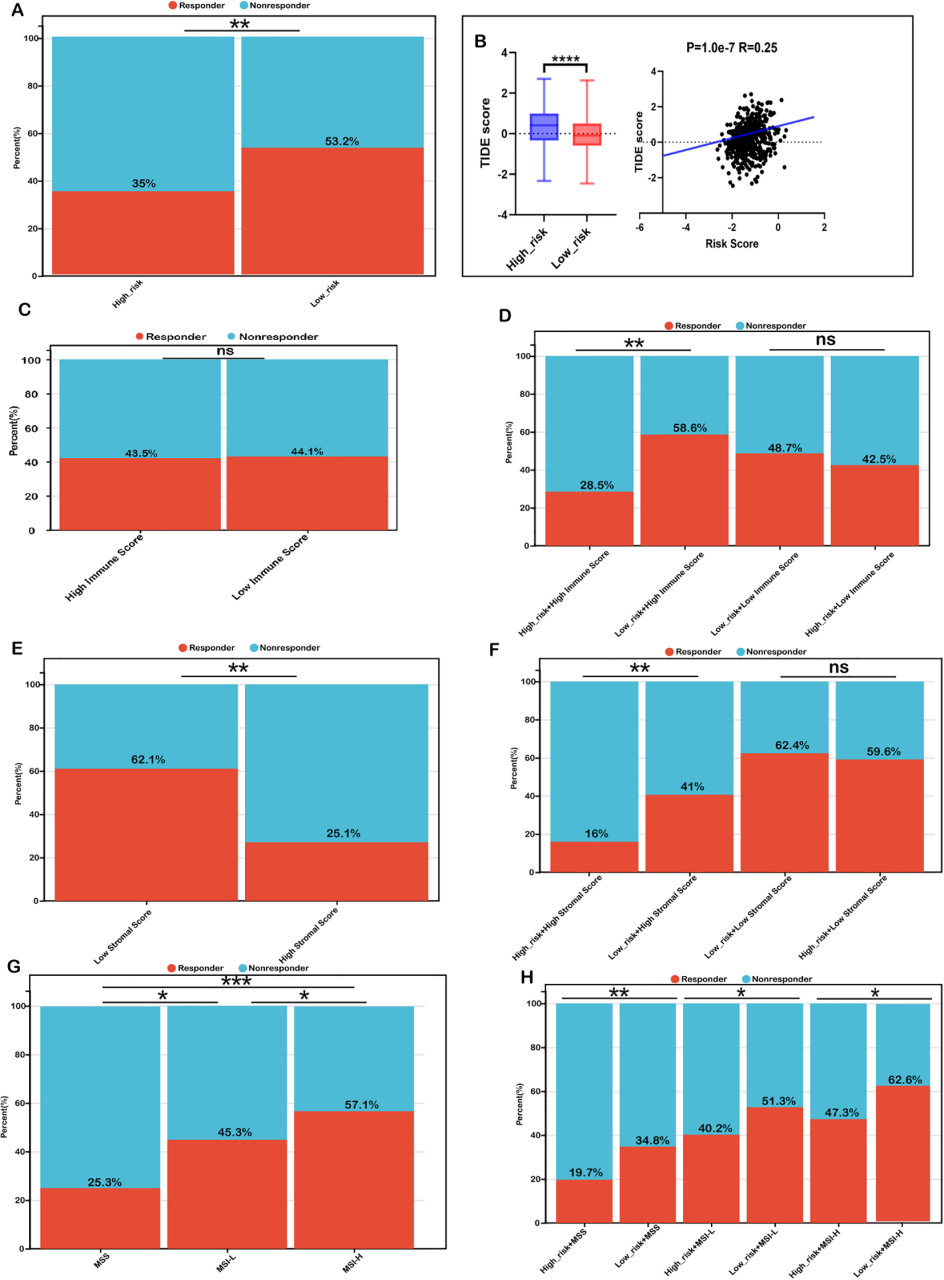
Risk score as a biomarker for predicting immunotherapy benefits in COADREAD. **(A)** Comparison of immunotherapy response proportions between low- and high-risk groups in the TCGA-COADREAD dataset. **(B)** TIDE scores for low- versus high-risk groups, including their correlation with the risk score. **(C)** TIDE-predicted immunotherapy response rates in groups stratified by low and high immune scores. **(D)** TIDE-predicted response rates for four groups defined by combined risk and immune scores. **(E)** TIDE-predicted response rates for groups stratified by low and high stromal scores. **(F)** TIDE-predicted response rates for four groups based on combined risk and stromal scores. **(G)** TIDE-predicted response rates in MSS, MSI-L, and MSI-H groups. **(H)** TIDE-predicted response rates in six groups, defined by combined risk score and microsatellite status (MSS: microsatellite stable; MSI-L: microsatellite instability-low; MSI-H: microsatellite instability-high). Significance: ns (not significant); ****P < 0.0001,***P < 0.001, **P < 0.01, *P < 0.05.

To validate these findings, we utilized the TIDE algorithm to predict immunotherapy responses in both low- and high-risk patient groups. The high-risk group exhibited a significantly lower response rate to immunotherapy (35%) compared to the low-risk group (53.2%) (**Figure 6A**). Additionally, the high-risk group had a significantly elevated TIDE score, which positively correlated with the risk score (**Figure 6B**). Given that a higher TIDE score is indicative of immune evasion and resistance to immune checkpoint inhibitors (ICIs), these results suggest that patients in the low-risk group, characterized by lower TIDE scores, are more likely to benefit from ICIs and achieve better survival outcomes following immunotherapy.

We further investigated whether combining the risk score with immune infiltration status improves the predictive accuracy of immunotherapy response. Immunotherapy response rates were similar between the high-immune (43.7%) and low-immune (44.1%) groups (**Figure 6C**). Within the low-immune subgroup, response rates in the low-risk (48.7%) and high-risk (42.5%) subgroups were comparable, indicating combined risk score and immune score was not better than immune score alone in patients with low immune infiltration. However, in the high-immune subgroup, the low-risk + high-immune group exhibited a significantly higher response rate (58.6%) compared to the overall high-immune group (43.5%), whereas the high-risk + high-immune group had a notably lower response rate (28.5%) (**Figure 6D**). These findings suggest that integrating the risk score with immune scores refines the prediction of immunotherapy response, particularly in COADREAD patients with high immune infiltration.

Similarly, we observed that the immunotherapy response rate was significantly higher in the low-stromal subgroup (62.1%) than in the high-stromal subgroup (25.1%) (**Figure 6E**). Within the low-stromal subgroup, the low-risk group had a slightly higher response rate (62.4%) than the high-risk group (59.6%), though the difference was not significant. However, in the high-stromal subgroup, the low-risk group demonstrated a significantly higher response rate (41.1%) compared to the high-risk group (16.6%) (**Figure 6F**), reinforcing the predictive value of integrating stromal scores with risk scores. Such a combination has the potential to enhance the predictive accuracy of immunotherapy responses, particularly in patients characterized by a stroma-rich tumor microenvironment.

Additionally, microsatellite instability-high (MSI-H) patients exhibited a markedly higher immunotherapy response rate (57.1%) compared to microsatellite stable (MSS, 25.1%) and MSI-low (MSI-L, 45.3%) subgroups (**Figure 6G**). Within each MSI category, low-risk patients consistently showed higher response rates than their high-risk counterparts, particularly in the MSI-H subgroup (62.6% vs. 47.3%) (**Figure 6H**). Collectively, these findings highlight that combining the risk score with immune scores, stromal scores, and MSI status may enhance the predictive accuracy of immunotherapy response in COADREAD patients, providing a potential framework for stratifying patients to optimize immunotherapy strategies.

Collectively, these findings underscore that the integration of risk scores with immune scores, stromal scores, and MSI status significantly may enhance the predictive accuracy for immunotherapy responses in COADREAD patients. This multi-dimensional approach provides a robust framework for patient stratification, ultimately aiding in the optimization of immunotherapy strategies.

### Mutation status of CRC patients in high−risk and low−risk groups

**Figure 7:**
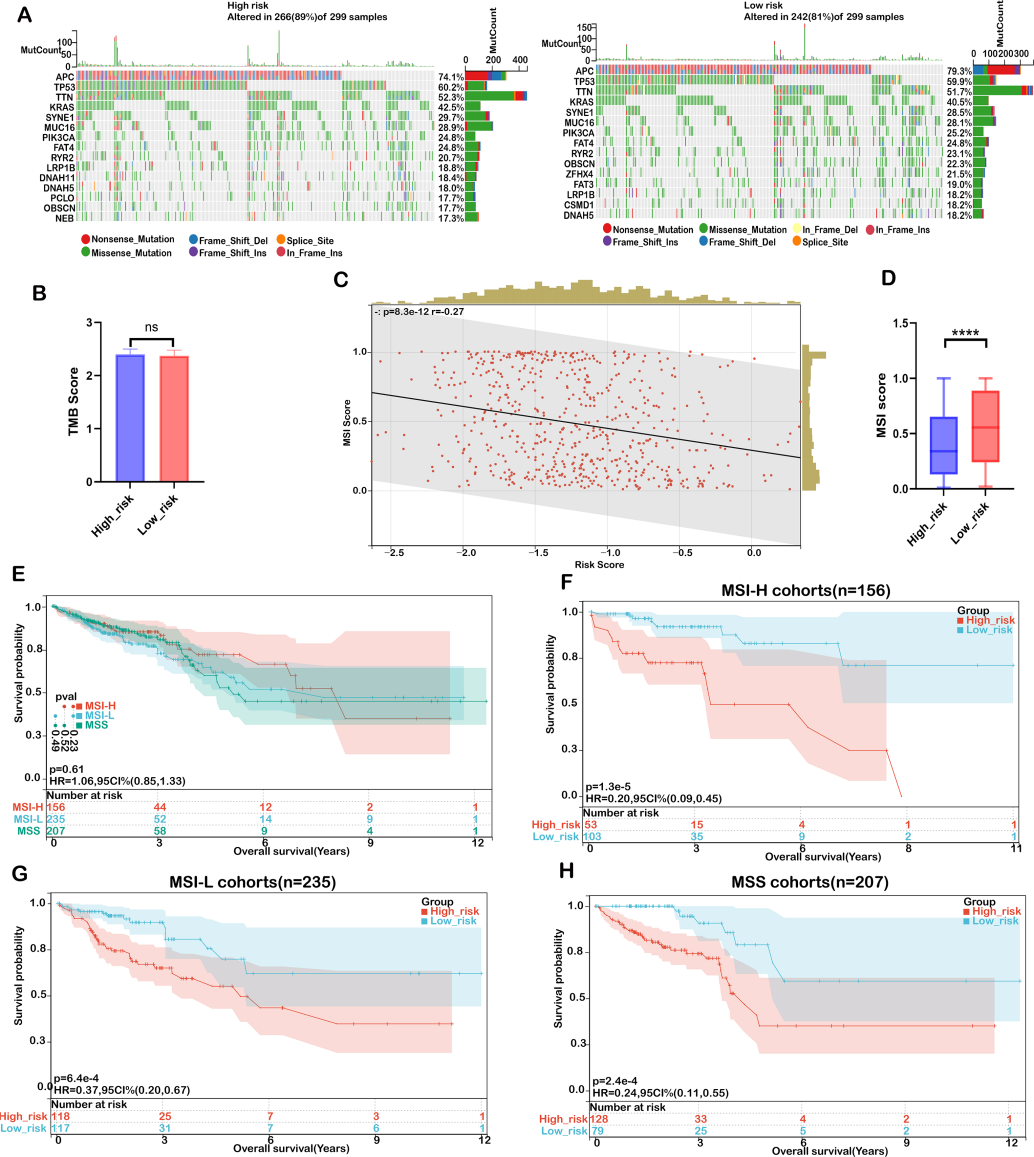
Mutation landscape in high- and low-risk COADREAD groups. **(A)** Mutation frequency for the top 15 genes in high- and low-risk groups. **(B)** Distribution of TMB scores in low- versus high-risk groups. **(C)** Correlation analysis between the risk score and the MSI gene expression signature. **(D)** Distribution of the MSI expression signature between risk groups. **(E)** Kaplan–Meier survival curves for patients classified as MSS, MSI-L, and MSI-H in the TCGA-COADREAD cohort. **(F)** Kaplan–Meier survival curves for patients in the MSI-H subgroup stratified by risk score. **(G)** Kaplan–Meier survival curves for the MSI-L subgroup based on risk score. **(H)** Kaplan–Meier survival curves for the MSS subgroup according to risk score. Significance: ns (not significant); ****P < 0.0001..

Accumulated mutations play a significant role in cancer development. Recent advances in genome sequencing have deepened our understanding of the somatic mutations that drive cancer, allowing us to pinpoint key oncogenes and unravel mutational processes. In our study, we characterized the mutation landscape of COADREAD by stratifying patients into high-risk and low-risk groups based on their risk scores. Notably, the most frequently mutated genes in both groups were APC, TP53, TTN, KRAS, MUC16, SYNE1, RYR2, FAT4, PIK3CA, and OBSCN (**Figure 7A**). However, a comparison of tumor mutational burden (TMB) between the two groups revealed no significant differences (**Figure 7B**), suggesting that TMB alone may not be a sufficient marker for risk stratification in COADREAD.

**Figure 8:**
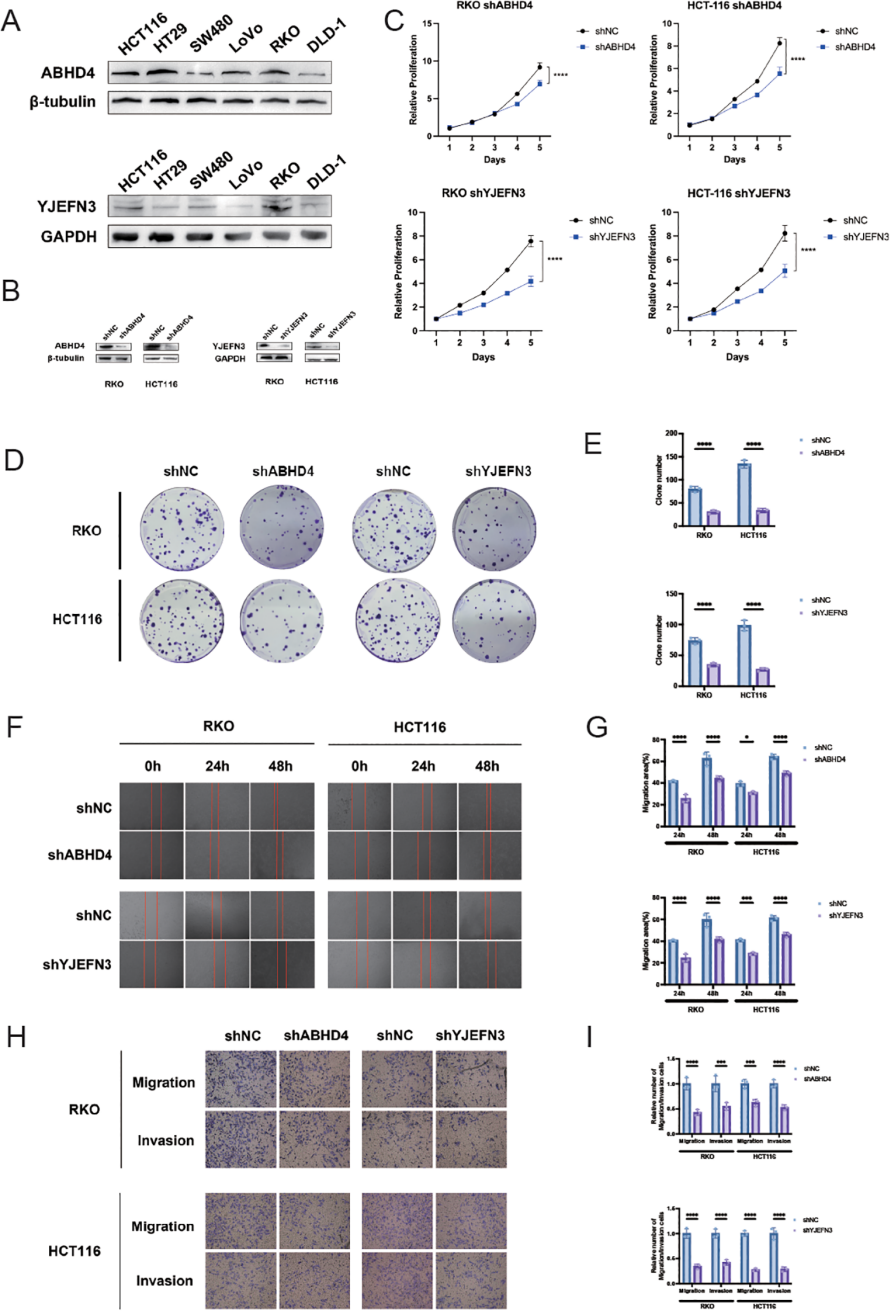
Knockdown of ABHD4 and YJEFN3 suppresses proliferation and migration of CRC cells *in vitro*. **(A)** Western blot analysis showing the expression levels of ABHD4 and YJEFN3 across six colorectal cancer (CRC) cell lines. **(B)** Confirmation of ABHD4 and YJEFN3 knockdown efficiency by Western blotting in stably transfected RKO and HCT116 cells. **(C)** Cell proliferation assessed using the CCK-8 assay. Statistical significance was evaluated using two-way ANOVA. **(D, E)** Representative images **(D)** and quantitative analysis **(E)** of colony formation assays demonstrating reduced clonogenic ability upon ABHD4 and YJEFN3 knockdown. **(F, G)** Representative images **(F)** and quantification **(G)** of wound healing assays showing impaired migratory capacity following ABHD4 and YJEFN3 silencing. **(H, I)** Representative images **(H)** and quantitative analysis **(I)** of Transwell migration assays further confirming reduced cell motility after ABHD4 and YJEFN3 knockdown. Data are presented as mean ± SD from at least three independent experiments. Significance:*P < 0.05, ***P < 0.001,****P < 0.0001.

Recognizing the limitations of relying solely on TMB, we expanded our analysis to include microsatellite instability (MSI), an important biomarker for predicting immunotherapy response in colorectal cancer. Our data indicated that MSI levels were significantly lower in the high-risk group compared to the low-risk group, and there was a robust negative correlation between the risk score and the MSI expression signature (**Figures 7C, D**). Although a higher MSI status (MSI-H) is generally associated with improved overall survival (OS), the observed differences did not reach statistical significance (**Figure 7E**). Importantly, across the MSI-H, MSI-L, and MSS subgroups, patients in the low-risk group consistently exhibited better OS than those in the high-risk group (**Figures 7F–H**).

Overall, these findings underscore the importance of integrating multiple biomarkers—specifically risk scores with MSI status—to enhance prognostic accuracy for COADREAD patients. This multifaceted approach provides a more robust strategy for patient stratification, thereby facilitating personalized treatment decisions and potentially improving clinical outcomes.

### Risk score predicts therapeutic benefits in COADREAD

To explore the potential of the mitochondrial lipid metabolism genes-related risk score as a predictive indicator for chemotherapy response, we assessed the half maximal inhibitory concentration (IC50) values for 198 drugs in patients from the TCGA cohort. By estimating these IC50 values, we aimed to determine whether the risk score could effectively differentiate drug sensitivity between high-risk and low-risk patient groups.

**Figure 9:**
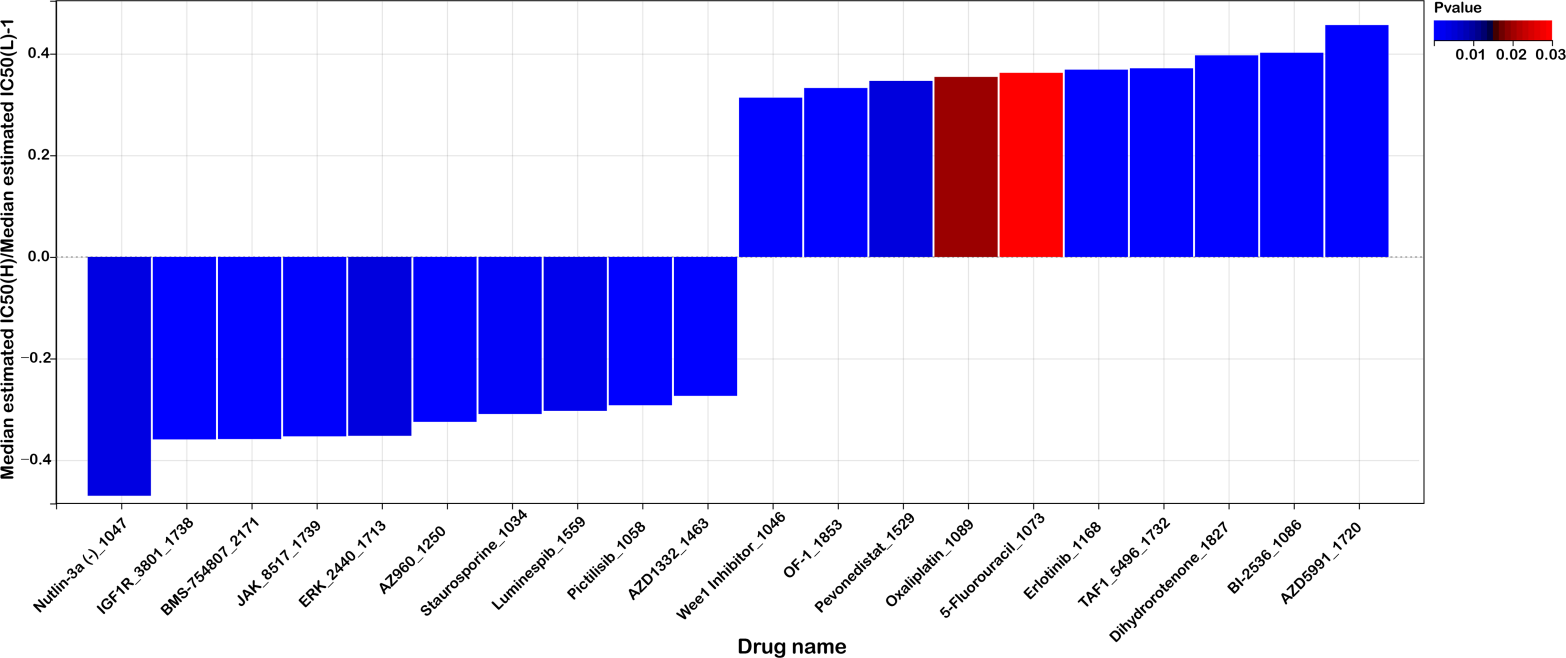
Risk score predicts drug sensitivity in colorectal cancer. A comparison of normalized IC50 values for the top 10 drugs between high- and low-risk groups, with significant differences observed (P < 0.01).

**Figure 9** shows the top 10 drugs with the most pronounced sensitivity differences between the high- and low-risk groups. These differences were statistically evaluated using both p-values and FDR-corrected q-values, all of which met the significance criteria (p < 0.05, q < 0.05; **Supplementary Table S1**), confirming the robustness and reliability of the observed drug sensitivity variations. Our analysis suggests that patients in the high-risk group may have increased sensitivity to specific chemotherapeutic agents. Notably, drugs such as Nutlin-3a(-)_1047, IGF1R_3801_1738, and BMS-754807_2171 demonstrated lower IC50 values in high-risk patients, suggesting that these individuals may respond more favorably to these treatments. These drugs target key pathways involved in cell cycle regulation and growth factor signaling, which may be more pronounced in tumors with altered mitochondrial lipid metabolism.

Conversely, the low-risk group appeared to be more responsive to other compounds, including Dihydrorotenone_1827, BI-2536_1086, and AZD5991_1720, as evidenced by their lower IC50 values in this subgroup. This differential sensitivity suggests that distinct molecular mechanisms might be driving tumor behavior in the low-risk cohort, and that these patients could benefit from tailored therapeutic strategies targeting these specific pathways.

Overall, our findings underscore the potential utility of the mitochondrial lipid metabolism genes-related risk score as a prognostic biomarker that not only stratifies patients based on clinical outcomes but also guides the selection of chemotherapeutic agents. By integrating the risk score with drug sensitivity data, personalized treatment strategies could be developed, potentially leading to improved clinical outcomes for COADREAD patients.

### Development of another prognostic nomogram integrating ABHD4, YJEFN3, and clinical parameters in CRC

To facilitate individualized prognostic assessment and guide clinical decision-making in colorectal cancer (CRC), we constructed a predictive nomogram by incorporating both prognostic model gene and clinical variables. Univariate and multivariate Cox regression analyses identified ABHD4, YJEFN3, pTNM stage, and age as statistically significant, suggesting their roles as independent prognostic indicators (**Supplementary Figures S7A, B**).

Based on these findings, we developed a nomogram model combining the expression levels of ABHD4 and YJEFN3 with key clinical characteristics, including pTNM stage and patient age, to estimate overall survival probabilities (**Supplementary Figure S5C**). The predictive performance of the nomogram was evaluated using time-dependent receiver operating characteristic (ROC) curves, which yielded area under the curve (AUC) values of 0.76, 0.79, and 0.80 for 1-, 3-, and 5-year overall survival (OS), respectively (**Supplementary Figure S8B**), indicating good discriminatory ability.

Furthermore, calibration plots showed excellent agreement between predicted and observed survival outcomes at each time point (1-, 3-, and 5-year), demonstrating the reliability of the model (**Supplementary Figure S5E**). The concordance index (C-index) of the nomogram reached 0.743 (95% CI: 0.696–0.791; p = 5.63 × 10&^-24^), further confirming its robust prognostic utility in CRC.

To further elucidate the functional relevance of ABHD4 and YJEFN3 as independent prognostic biomarkers, we explored their expression patterns in different cellular compartments of the colorectal cancer tumor microenvironment using the TISCH2 single-cell transcriptomic database.

Interestingly, ABHD4 showed higher expression in stromal cell populations, including fibroblasts and endothelial cells, compared to both tumor and immune cells (**Supplementary Figure S9**). This suggests that ABHD4 may exert its tumor-promoting effects through modulating the stromal components of the TME, such as extracellular matrix remodeling or angiogenesis, rather than direct oncogenic activity in tumor cells. Such stromal expression may still correlate with poor prognosis due to its influence on tumor invasion, immune evasion, or therapy resistance.

In contrast, YJEFN3 expression was predominantly enriched in malignant epithelial (tumor) cells, whereas its expression was markedly lower in immune cells (**Supplementary Figure S10**). This tumor cell-specific overexpression supports its role as a tumor-intrinsic factor potentially contributing to cancer cell survival and aggressiveness, consistent with its identification as a high-risk prognostic gene.

These distinct expression landscapes highlight that ABHD4 and YJEFN3 may contribute to CRC progression through different cellular mechanisms within the TME, emphasizing the importance of considering cell-type specificity in biomarker interpretation and therapeutic targeting.

### Functional characterization of ABHD4 and YJEFN3 in CRC cell proliferation and migration *in vitro*

To deepen our understanding of the clinical and biological implications of the prognostic model, ABHD4 and YJEFN3 were selected from the 13-gene signature for further experimental validation. This selection was guided by three key criteria: (1) both genes exhibited among the highest positive coefficients and hazard ratios in multivariate Cox regression analysis, underscoring their strong prognostic relevance; (2) they demonstrated statistically significant associations in both univariate and multivariate Cox models, supporting their roles as independent prognostic factors; and (3) their functional roles in colorectal cancer remain largely unexplored, presenting a valuable opportunity to uncover novel mechanisms driving CRC progression. To this end, we first examined the endogenous expression levels of ABHD4 and YJEFN3 in a panel of six CRC cell lines using Western blotting (**Figure 8A**). The results revealed detectable expression in multiple cell lines, providing a foundation for subsequent functional assays.

To evaluate the functional significance of these genes, we constructed stable knockdown models using lentivirus-mediated short hairpin RNAs targeting ABHD4 and YJEFN3 in RKO and HCT116 cell lines, respectively (RKO/shABHD4, HCT116/shABHD4; RKO/shYJEFN3, HCT116/shYJEFN3) (**Figure 8B**). Successful knockdown was confirmed at the protein level via Western blot analysis.

Functional assays demonstrated that silencing of ABHD4 or YJEFN3 markedly suppressed CRC cell viability, as assessed by CCK-8 and colony formation assays (**Figures 8C–E**). Moreover, transwell and wound healing assays revealed that the knockdown of either gene significantly impaired the migratory and invasive capabilities of CRC cells (**Figures 8F–I**), suggesting that both ABHD4 and YJEFN3 are positively associated with tumor cell aggressiveness.

Collectively, these findings indicate that ABHD4 and YJEFN3 may function as oncogenic regulators in CRC, and their inhibition could serve as a potential therapeutic strategy to limit CRC progression.

## Discussion

Colorectal cancer (CRC) ranks as one of the most common and deadly cancers globally, being the third most diagnosed and the second leading cause of cancer death (20). Prognosis in CRC is heavily dependent on the stage at diagnosis, with an overall 5-year survival rate of approximately 65% (21). Metabolic reprogramming, especially in lipid metabolism, is a hallmark of malignancy that critically shapes the TME and influences cancer progression as well as treatment outcomes (22). In recent decades, immunotherapy has revolutionized the management of advanced cancers, and alterations in lipid metabolism have emerged as important modulators of the immune landscape and responsiveness to such therapies (23). However, few studies have explored the prognostic value of mitochondrial lipid metabolism-related genes in CRC, particularly in the context of developing predictive models. This study aims to address this gap by identifying mitochondrial lipid metabolism-related genes that may serve as prognostic biomarkers, thereby supporting early intervention and personalized treatment strategies for high-risk CRC patients.

Currently, many biomarkers were applied for prognostic prediction of CRC, such as ACAT2,ATG7, MAPK1, but most of them are studied for a single biomarker (24–26). Increasing evidences indicated that prognostic model constructed by multi-genes as a prognostic index was more comprehensive and effective than single gene in kinds of malignancies. For instance, Zheng H et al. constructed a prognostic signature for colorectal cancer (CRC) that was specifically based on cancer-associated fibroblast (CAF) markers (27). Zhang et al. constructed a neurotransmitter receptor-related gene signature as potential prognostic and therapeutic biomarkers in colorectal cancer (28). As the dysfunction of mitochondrial lipid metabolism have been associated with cancer, we constructed a CRC prognostic model based on mitochondrial lipid metabolism-related genes that could be used to predict the prognosis and efficacy of immunotherapy in patients with CRC.

In our research, we identified mitochondrial lipid metabolism-related genes by analyzing data from the MSigDB and TCGA databases. Through univariate Cox and LASSO regression analyses, we narrowed down to 13 pivotal genes. Among these, genes with positive coefficients (ABHD4, ABHD8, YJEFN3, CRYAB, and HSPA1A) were found to be risk factors, suggesting that their increased expression is associated with worse prognoses in colorectal cancer patients. Conversely, genes with negative coefficients (HDHD5, PNPLA4, GK5, CPT2, MAPK1, ATG7, HDAC3, and ACAT2) were identified as protective factors, with higher expression correlating with improved survival outcomes. Several of these genes are already known to play significant roles in CRC progression, reinforcing their potential as biomarkers.

To validate our predictive model, we conducted both internal and external evaluations. The internal validation, using ROC analysis, revealed strong sensitivity and specificity (AUC = 0.72). Kaplan-Meier survival analysis indicated that high-risk patients had significantly worse survival outcomes. External validation with GEO datasets confirmed these findings, showing that low-risk patients had better overall survival. These results underscore the reliability of mitochondrial lipid metabolism-related genes as prognostic markers in CRC. In our study, head-to-head ROC analyses further highlighted the robustness of our 13-gene signature in prognostic prediction for CRC (**Figure 2G**). Specifically, our model yielded the highest AUC value (0.72), exceeding those of previously reported metabolism-related models, including signatures based on nucleotide metabolism, general metabolic genes, tryptophan metabolism, mRNAsi-related metabolic risk scores, and hypoxia- or lipid metabolism–associated genes (29–33). This superior performance suggests that our signature may capture critical metabolic alterations that are more closely linked to CRC progression and patient outcomes. Therefore, it provides a more reliable tool for prognostic stratification and potentially facilitates personalized therapeutic decision-making compared with existing metabolic models.

Further examination of the differentially expressed genes (DEGs) between the high- and low-risk groups highlighted significant enrichment in pathways related to extracellular matrix (ECM) organization, particularly those involving extracellular matrix structural constituents. This finding is consistent with previous studies that have established ECM accumulation as a characteristic feature of aggressive tumor behavior, often linked to poor prognosis in various types of cancer (34–36). The tumor microenvironment, shaped by its complex interactions with immune cells and stromal components such as fibroblasts, plays a critical role in the advancement of colorectal cancer (37, 38). In the TME, fibroblasts are transformed into cancer-associated fibroblasts (CAFs), which are abundant in both primary and metastatic tumors. CAFs are known for their remarkable adaptability and resilience, significantly influencing cancer progression through interactions with other TME components (39, 40). The matrisome, encompassing genes that encode core ECM proteins and structural elements, is essential for understanding cancer biology (41). For instance, Chao Huang developed a novel prognostic matrisome-related gene signature for head and neck squamous cell carcinoma (42). Consistent with these findings, our analysis identified a strong positive correlation between the risk score and the expression of CAF, ECM, and matrisome-related genes. Furthermore, we observed a positive correlation between the risk score and stromal score, along with a negative correlation with tumor purity, indicating that stromal cell infiltration is increased in the TME of high-risk colorectal cancer patients. These results reinforce previous studies that have highlighted the prognostic importance of stromal components in colorectal cancer progression.

Immune cells play a crucial role in the tumor microenvironment, significantly influencing both tumor progression and therapeutic responses. Recent research has demonstrated that different TME phenotypes are associated with varying immunotherapeutic outcomes and clinical prognoses (43–45). One of the key benefits of immunotherapy is its ability to stimulate memory CD8+ T cells, which offer long-term protection against tumor metastasis and recurrence (46–48). Emerging studies suggest that these TME phenotypes are also linked to differences in survival rates and immune therapy responses (49, 50). Given the central role of immune cells in the TME and their impact on treatment effectiveness, we investigated the differences in immune cell composition between the high- and low-risk groups.

In this study, we employed the CIBERSORT algorithm, a deconvolution-based method that estimates the relative abundance of immune cell subsets from gene expression data, to characterize the immune landscape across different risk groups. Our findings revealed distinct immune cell distribution patterns between high- and low-risk samples, suggesting potential immunological mechanisms underlying tumor progression.

In high-risk CRC samples, M0 macrophages and Treg cells were significantly enriched, indicating an immunosuppressive TME that may facilitate tumor progression. Positive correlations with naive CD4+ T cells, memory B cells, CD8+ T cells, and activated NK cells suggest immune dysfunction or exhaustion despite their typical antitumor roles. Activated mast cell enrichment may further shape the local immune milieu.

In contrast, low-risk samples showed higher levels of resting and activated memory CD4+ T cells, plasma cells, and dendritic cells, reflecting enhanced immune memory and antigen presentation. Overall, high-risk tumors appear linked to immunosuppression, while low-risk tumors exhibit stronger immune activation. These findings highlight complex immune regulation in CRC and suggest implications for optimizing immunotherapy.

Monoclonal antibodies targeting immune checkpoint molecules have marked a significant advancement in cancer treatment (51). TIDE scoring is a crucial predictor of immunotherapy response, with higher TIDE scores correlating with lower response rates. In our study, an increase in the risk score was associated with a significant decline in immunotherapy response, with the low-risk group exhibiting a response rate of 53.2% compared to only 35% in the high-risk group, thereby underscoring the predictive value of the risk score.

When patients were stratified by immune score, overall response rates did not significantly difference between high and low immune score groups (**Figure 6C**; low risk, 43.5% vs. high risk, 44.1%). Within the low immune score subgroup, the differences in response between high- and low-risk patients were not statistically significant (low risk, 48.7% vs. high risk, 42.5%). However, in the high immune score subgroup, the low-risk group achieved a markedly higher response rate (58.6%) than the high-risk group (28.5%), suggesting that although a high immune score generally predicts favorable outcomes, its benefit is substantially diminished in patients with a high risk score.

Similarly, stromal score analysis demonstrated that patients in the low-stromal group had a significantly higher response rate (62%) compared to those in the high-stromal group (25%), indicating that a low-stromal environment is more conducive to immunotherapy. Further subgroup analysis revealed that within the low-stromal group, low-risk patients had a response rate of 62.4% versus 59.6% in high-risk patients, whereas in the high-stromal group, the response rates were 41% and 16% for low- and high-risk patients, respectively. These data imply that risk and stromal scores exert a synergistic effect on immunotherapy response.

A growing body of clinical evidence indicates that while anti-PD-1 and anti-PD-L1 therapies yield favorable outcomes in dMMR/MSI-H cancers, they are less effective in cold pMMR/MSS colorectal cancer. This association is likely due to MSI’s role in generating neoantigens that enhance antitumor immune responses, thereby serving as a robust predictor for PD-L1 therapy efficacy (52, 53). Our MSI subgroup analysis further revealed marked differences in immunotherapy response between MSI-H and MSS/MSI-L groups. Notably, risk stratification within each MSI category consistently showed that patients with lower risk scores had higher response rates compared to their high-risk counterparts: 51.3% versus 40.2% in the MSS subgroup, 62.6% versus 47.3% in the MSI-L subgroup, and 34.8% versus 19.7% in the MSI-H subgroup. These findings suggest that, regardless of MSI status, patients classified as low risk derive greater benefit from immunotherapy, whereas those with high risk scores demonstrate considerably reduced responses.

Our analysis of the mutation landscape in COADREAD shows that tumor mutational burden (TMB) alone does not effectively stratify patients, as no significant differences were observed in TMB or its association with the risk score. While TMB is prognostic in other cancers, it appears insufficient as an independent marker in CRC. By contrast, integrating the risk score with microsatellite instability (MSI) improved prognostic accuracy. Patients in the low-risk group consistently showed better survival across MSI subgroups, and the negative correlation between risk score and MSI further highlights their complementary value. These findings suggest that combining MSI status with the risk model offers a more robust prognostic framework and may help refine personalized treatment strategies in CRC.

Our findings suggest that the risk score may guide drug selection for COADREAD patients by revealing distinct sensitivity patterns between risk groups. High-risk patients showed greater sensitivity to agents targeting cell cycle and growth factor pathways (e.g., Nutlin-3a, IGF1R inhibitors, BMS-754807), while low-risk patients were more responsive to drugs affecting mitochondrial function, PLK1 inhibition, and apoptosis (e.g., Dihydrorotenone, BI-2536, AZD5991). These results highlight potential therapeutic vulnerabilities that could inform risk-adapted treatment strategies.

In this study, we constructed a prognostic nomogram incorporating ABHD4, YJEFN3, and key clinical parameters to improve individualized survival prediction in colorectal cancer (CRC). Both genes, along with pTNM stage and age, were identified as independent prognostic factors through univariate and multivariate Cox analyses. The nomogram showed good predictive accuracy, with AUC values above 0.75 for 1-, 3-, and 5-year overall survival and a C-index of 0.743, supporting its clinical utility.

Recent studies have highlighted the regulatory role of ABHD4 in lipid metabolism, particularly through its catalytic activity in converting NAPE and lyso-NAPE into GP-NAE, intermediates in the biosynthesis of bioactive N-acyl ethanolamines (NAEs) (54). Given the importance of lipid reprogramming in cancer, ABHD4 may promote CRC progression through lipid signaling modulation. Single-cell analysis showed its expression is enriched in stromal cells (fibroblasts, endothelial cells), implicating a role in remodeling the tumor microenvironment. Functional assays confirmed that ABHD4 knockdown inhibited CRC cell proliferation, migration, and invasion. Together, these findings indicate that ABHD4 may act as a tumor-promoting factor in CRC via both cell-intrinsic and stromal mechanisms, warranting further investigation as a prognostic or therapeutic target.

YJEFN3 (YjeF N-terminal domain-containing protein 3) has been identified as a tumor-associated antigen in prostate adenocarcinoma (PRAD), where its overexpression and mutations are linked to poor prognosis and altered immune cell infiltration (55). Its strong association with antigen-presenting cells suggests a potential role in modulating the tumor immune microenvironment (55). Although its role in CRC remains unclear, the immunogenicity and prognostic relevance observed in PRAD indicate that YJEFN3 may similarly contribute to CRC progression and immune regulation.

In our study, YJEFN3 was identified as a potential oncogenic driver in CRC. Knockdown experiments showed that silencing YJEFN3 suppressed cell proliferation, colony formation, migration, and invasion, confirming its role in sustaining malignant phenotypes. Single-cell analysis revealed predominant expression in tumor epithelial cells, supporting a tumor-intrinsic function. Together with its classification as a high-risk gene in Cox regression, these findings suggest that YJEFN3 contributes to CRC progression and may serve as a prognostic biomarker and therapeutic target.

Although our *in vivo* experiments provided preliminary support for the prognostic relevance of the identified genes, the validation was not comprehensive. In particular, detailed histological and molecular assessments (e.g., H&E staining, Ki-67) were not conducted, which restricts the extent to which the *in vivo* findings can substantiate the functional roles of the risk model genes. To address these shortcomings, future studies will establish orthotopic CRC xenograft models to better recapitulate the native tumor microenvironment, increase the animal sample size, and incorporate systematic evaluations, including histopathological analysis, molecular assays, and metastatic indicators. Such comprehensive validation will provide deeper insights into the biological mechanisms underlying the prognostic signature and further enhance its translational significance.

The translational significance of our study lies in the establishment of a prognostic model based on multiple mitochondrial lipid metabolism–related genes, which may provide improved prognostic value compared to traditional single-gene approaches. This model has the potential to stratify patients with COADREAD into high- and low-risk groups and may serve as a useful tool to complement existing diagnostic and prognostic methods. Its predictive relevance was further supported by associations with chemotherapy sensitivity, immunotherapy response, and immune cell infiltration, suggesting possible applications in guiding more personalized treatment strategies. In addition, silencing ABHD4 and YJEFN3 suppressed CRC cell proliferation and motility, validating their role in tumor progression and suggesting their potential as therapeutic targets with clinical relevance. While these findings underscore the potential biological and clinical relevance of our work, further *in vivo* and clinical validation will be necessary before translation into routine practice.

## Conclusions

Our findings present a novel risk score model based on genes associated with mitochondrial lipid metabolism. This score is closely linked to the tumor microenvironment and immune cell infiltration in COADREAD patients. When combined with stromal and immune scores, or MSS/MSI status, the model more accurately predicts immunotherapy response than any single metric alone. Regarding drug sensitivity, high-risk patients showed greater responsiveness to Nutlin-3a (-), IGF1R inhibitor (IGF1R_3801_1738), and BMS-754807, whereas low-risk patients were more responsive to Dihydrorotenone, BI-2536, and AZD5991. Overall, our mitochondrial lipid metabolism-related risk model may serve as a robust prognostic biomarker to facilitate personalized treatment strategies in COADREAD.

## Data Availability

The datasets presented in this study can be found in online repositories. The names of the repository/repositories and accession number(s) can be found in the article/**Supplementary Material**.
