# Supplementary material for: A mitochondrial lipid metabolism–related gene signature predicts prognosis and immune landscape in colorectal cancer
Source: Front Immunol. 2025 Nov 10;16:1669678. doi: 10.3389/fimmu.2025.1669678 (PMC12640859; doi:10.3389/fimmu.2025.1669678)
Supplement: Supplementary file 1 [file DataSheet1.pdf]

A Mitochondrial Lipid Metabolism–Related Gene Signature Predicts Prognosis and Immune Landscape in Colorectal Cancer

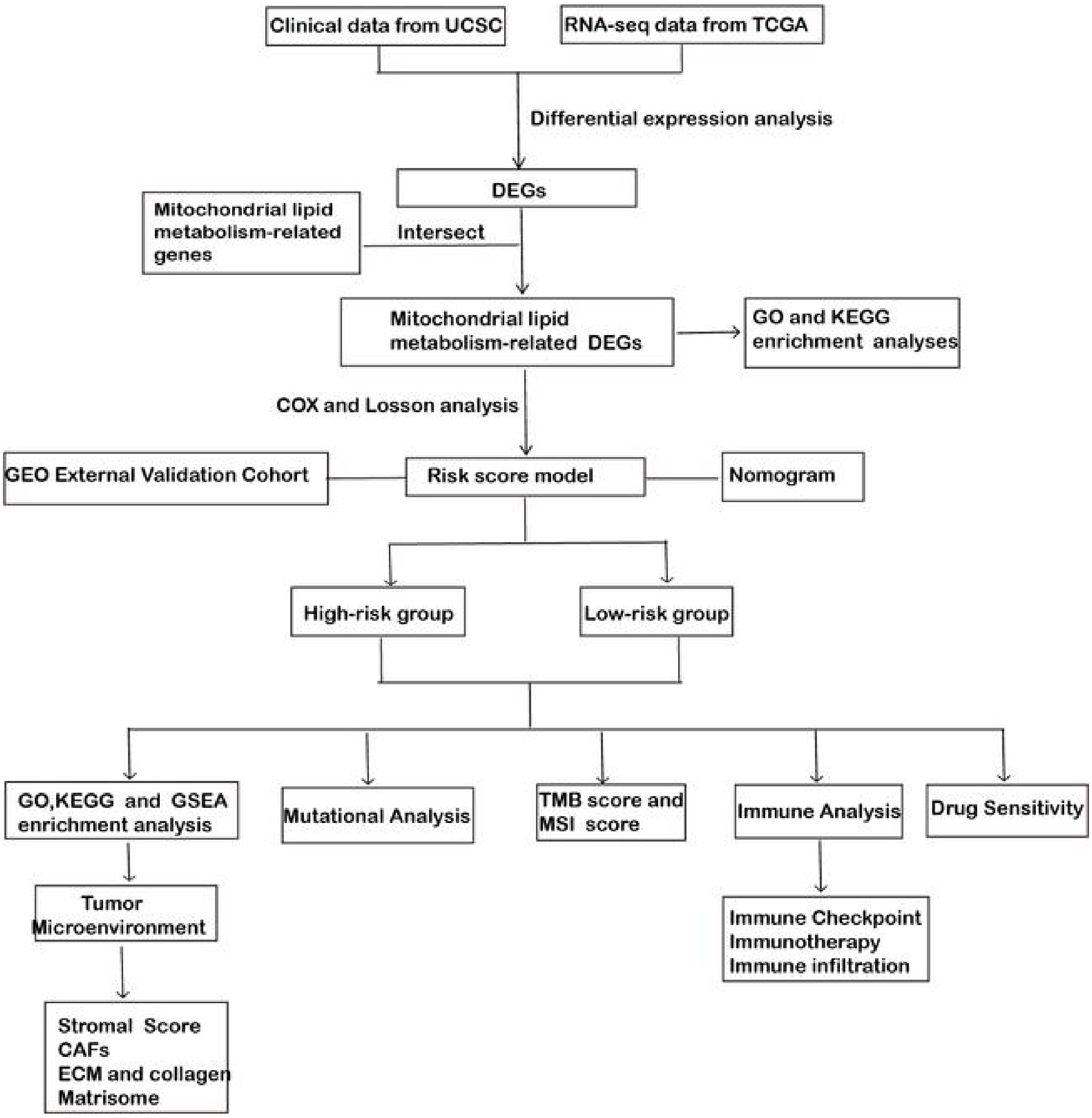

Figure S1. Workflow Diagram: A schematic overview of the study design and analytical steps.

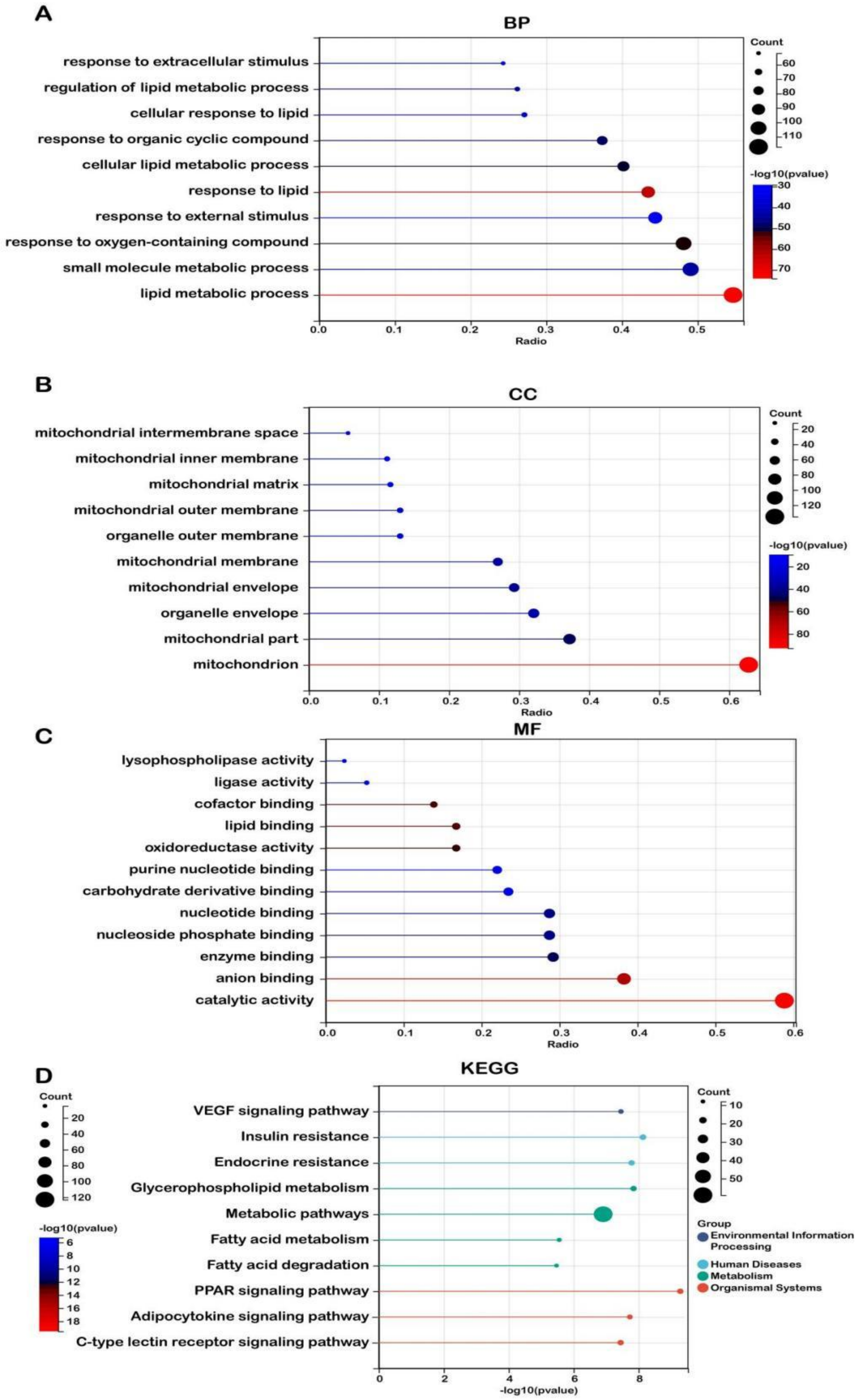

A
BP
response to extracellular stimulus
regulation of lipid metabolic process
cellular response to lipid
response to organic cyclic compound
cellular lipid metabolic process
response to lipid
response to external stimulus
response to oxygen-containing compound
small molecule metabolic process
lipid metabolic process
Radio
Count
-log10(pvalue)
B
CC
mitochondrial intermembrane space
mitochondrial inner membrane
mitochondrial matrix
mitochondrial outer membrane
organelle outer membrane
mitochondrial membrane
mitochondrial envelope
organelle envelope
mitochondrial part
mitochondrion
Radio
Count
-log10(pvalue)
C
MF
lysophospholipase activity
ligase activity
cofactor binding
lipid binding
oxidoreductase activity
purine nucleotide binding
carbohydrate derivative binding
nucleotide binding
nucleoside phosphate binding
enzyme binding
anion binding
catalytic activity
Radio
D
KEGG
Count
-log10(pvalue)
VEGF signaling pathway
Insulin resistance
Endocrine resistance
Glycerophospholipid metabolism
Metabolic pathways
Fatty acid metabolism
Fatty acid degradation
PPAR signaling pathway
Adipocytokine signaling pathway
C-type lectin receptor signaling pathway
-log10(pvalue)
Count
Group
Environmental Information Processing
Human Diseases
Metabolism
Organismal Systems

Figure S2. Identification of DEGs related to mitochondrion lipid metabolism and functional enrichment analysis in CRC. The GO analysis of 220 mitochondrial-related DEGs, including**(A)** biological process (BP), **(B)** cellular component (CC), and **(C)**molecular function (MF). **(D)** The KEGG analysis of 220 mitochondrial-related DEGs.

Gene Symbol	Full Name	Function/Role
ABHD4	Abhydrolase Domain Containing 4	Involved in lipid metabolism; may participate in phospholipid remodeling and signaling
ABHD8	Abhydrolase Domain Containing 8	Implicated in lipid processing and metabolic regulation
HDHD5	Haloacid Dehalogenase-Like Hydrolase Domain Containing 5	Putative hydrolase with potential roles in cellular metabolism and lipid processing
PNPLA4	Patatin-Like Phospholipase Domain Containing 4	Functions in lipid catabolism and remodeling of lipid molecules
GK5	Glycerol Kinase 5	Involved in glycerol metabolism and energy homeostasis
CPT2	Carnitine Palmitoyltransferase 2	Key enzyme in mitochondrial fatty acid β-oxidation, facilitating fatty acid transport
YJEFN3	YjeF N-Terminal Domain Containing 3	Function not well characterized; may play a role in metabolic processes
CRYAB	Crystallin Alpha B	Small heat shock protein involved in stress response and maintenance of cytoskeletal integrity
HSPA1A	Heat Shock Protein Family A (Hsp70) Member 1A	Acts as a molecular chaperone assisting in protein folding and cellular stress response
MAPK1	Mitogen-Activated Protein Kinase 1 (ERK2)	Central component in cell signaling pathways regulating proliferation, differentiation, and survival
ATG7	Autophagy Related 7	Essential for autophagy, contributing to the formation of autophagosomes
HDAC3	Histone Deacetylase 3	Plays a critical role in chromatin remodeling and transcriptional regulation
ACAT2	Acetyl-CoA Acetyltransferase 2	Involved in cholesterol metabolism and overall lipid homeostasis

Figure S3. The information of 13 prognosis-related genes

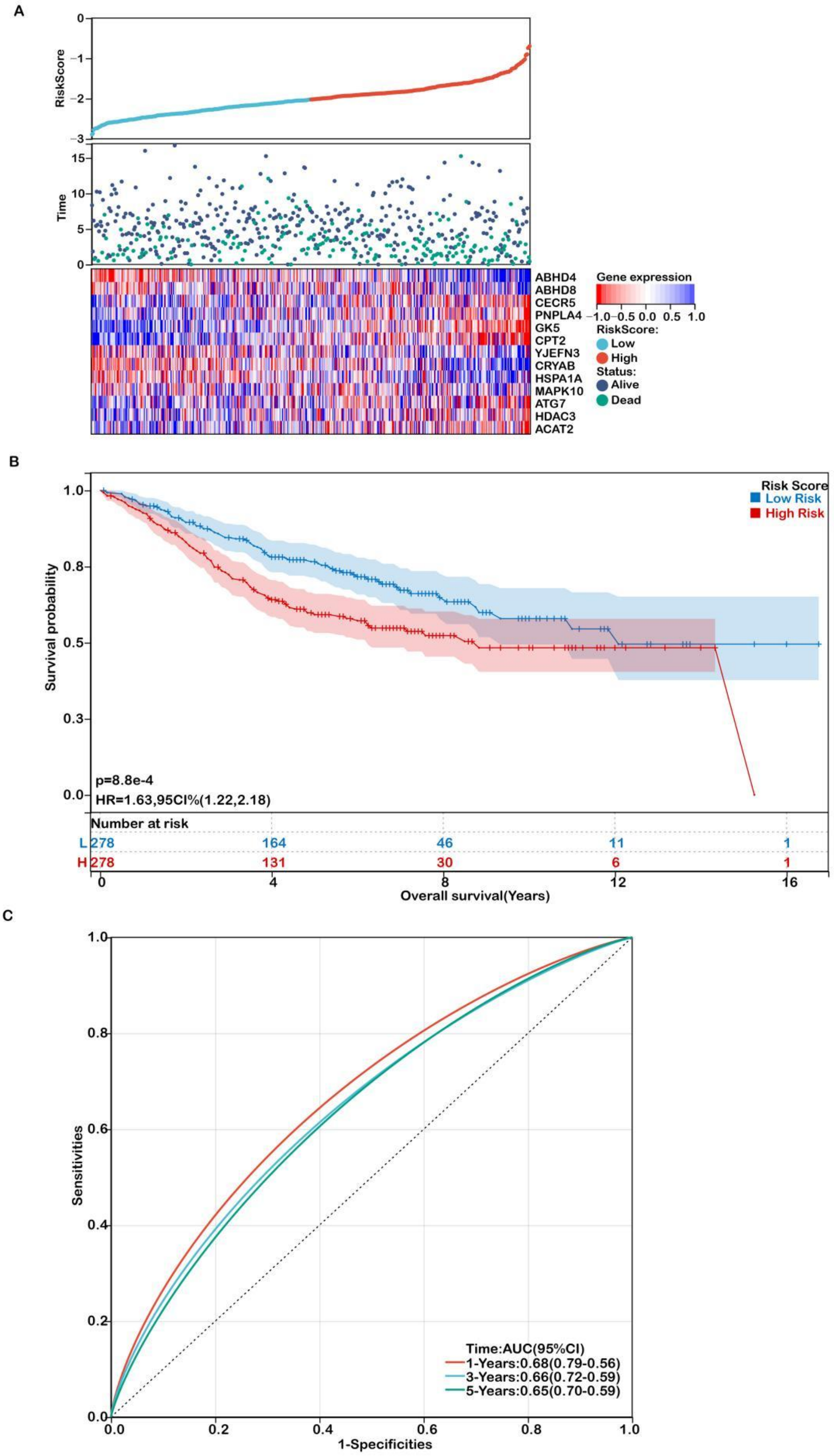

Figure S4. Validation of the Prognostic Model in an Independent Cohort

(A) Distribution of risk scores, survival status (purple indicates deceased, green indicates alive), and gene expression of the 13 model genes in the GSE39582 validation cohort. **(B)** Kaplan-Meier survival curves for overall survival in high- versus low-risk groups. **(C)** ROC curves for 1-, 3-, and 5-year overall survival predictions in the validation cohort.

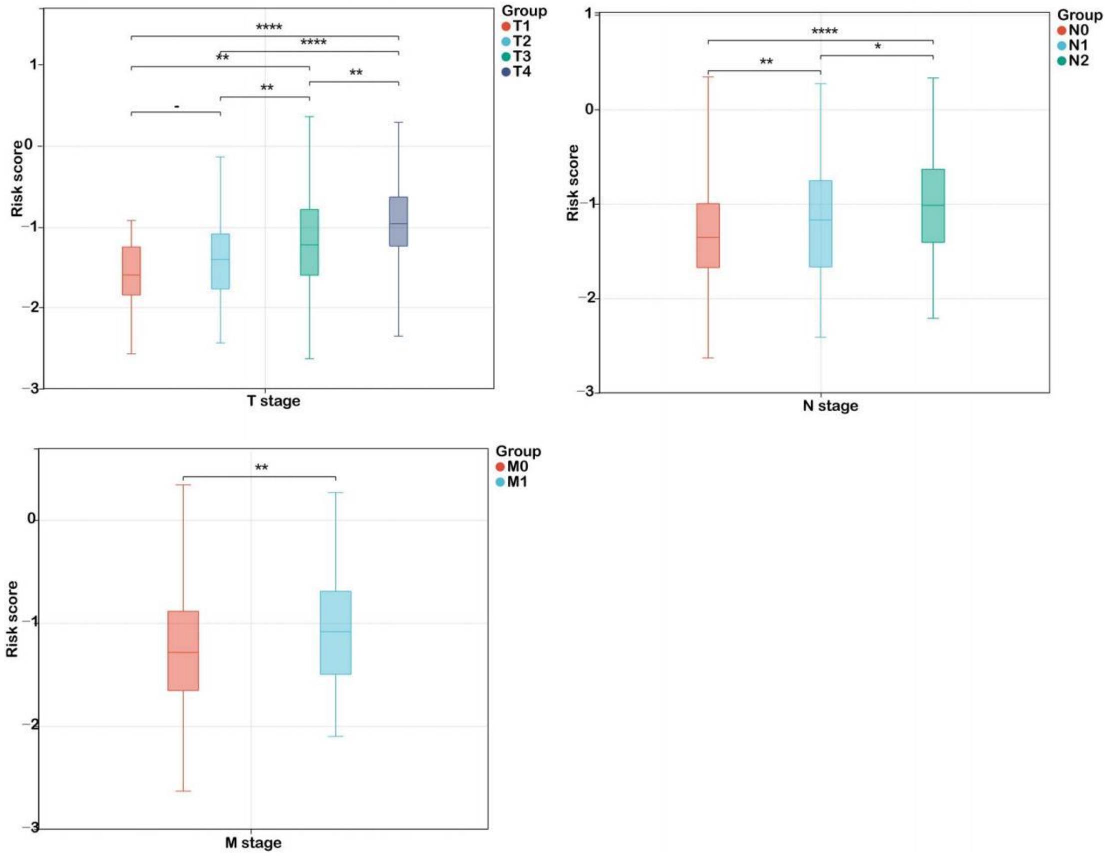

Figure S5. The relationships between the risk score and clinical characteristics of CRC patients. (A)T stage (B)N stage (C)M stage

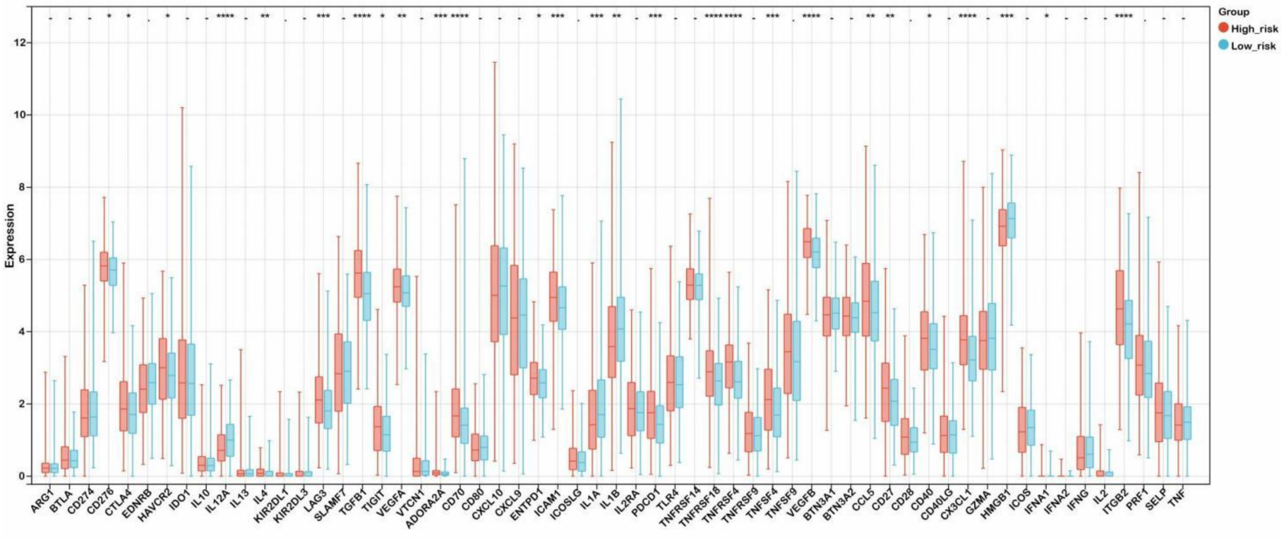

Figure S6. Quantitative assessment of immune cell subpopulations in high- and low-risk groups using the CIBERSORT algorithm.

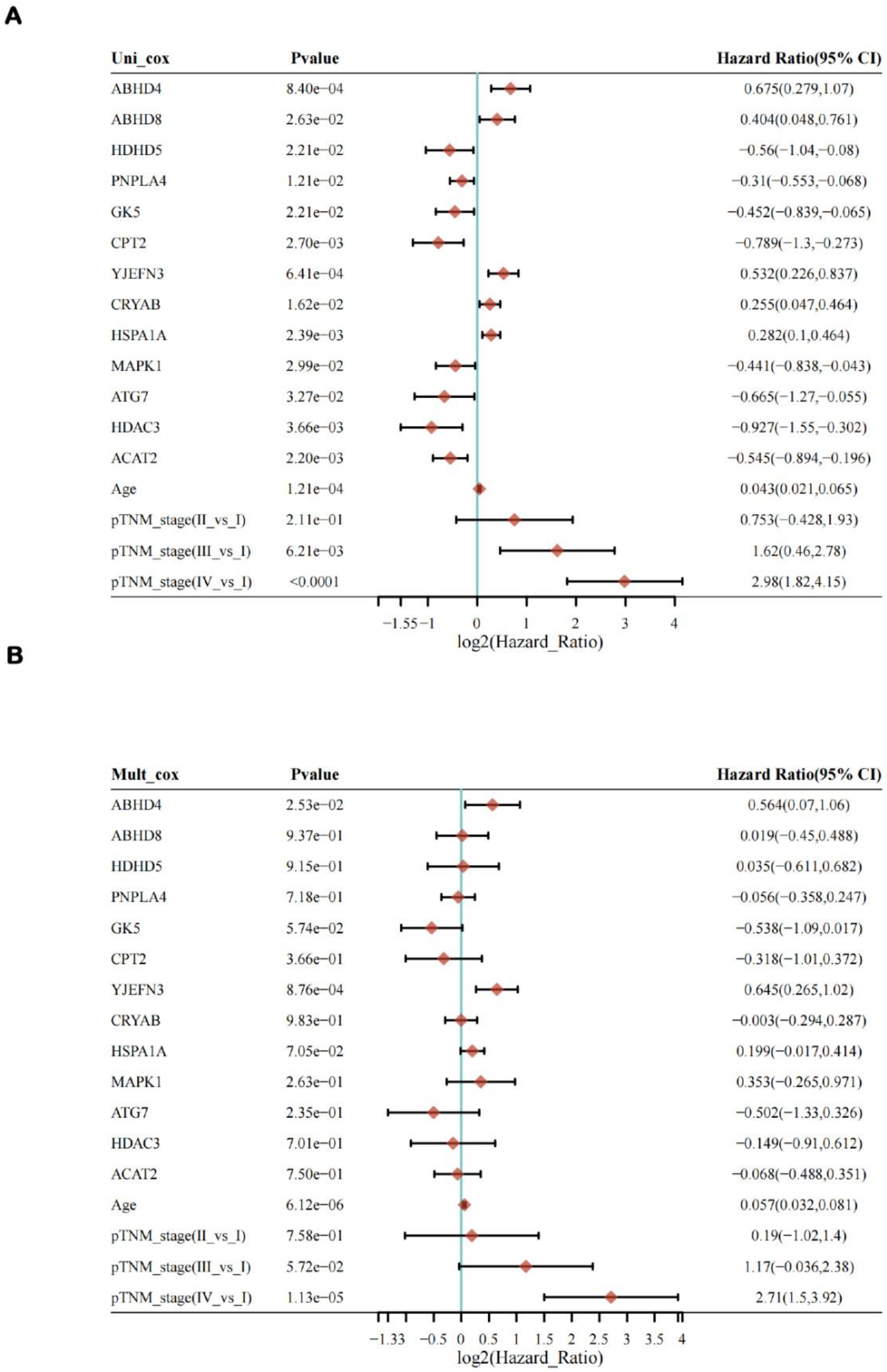

Figure S7. Identification of independent prognostic biomarkers in COADREAD. (A) Univariate Cox regression analysis of candidate genes. (B) Multivariate Cox regression confirming two genes as independent predictors of patient prognosis.

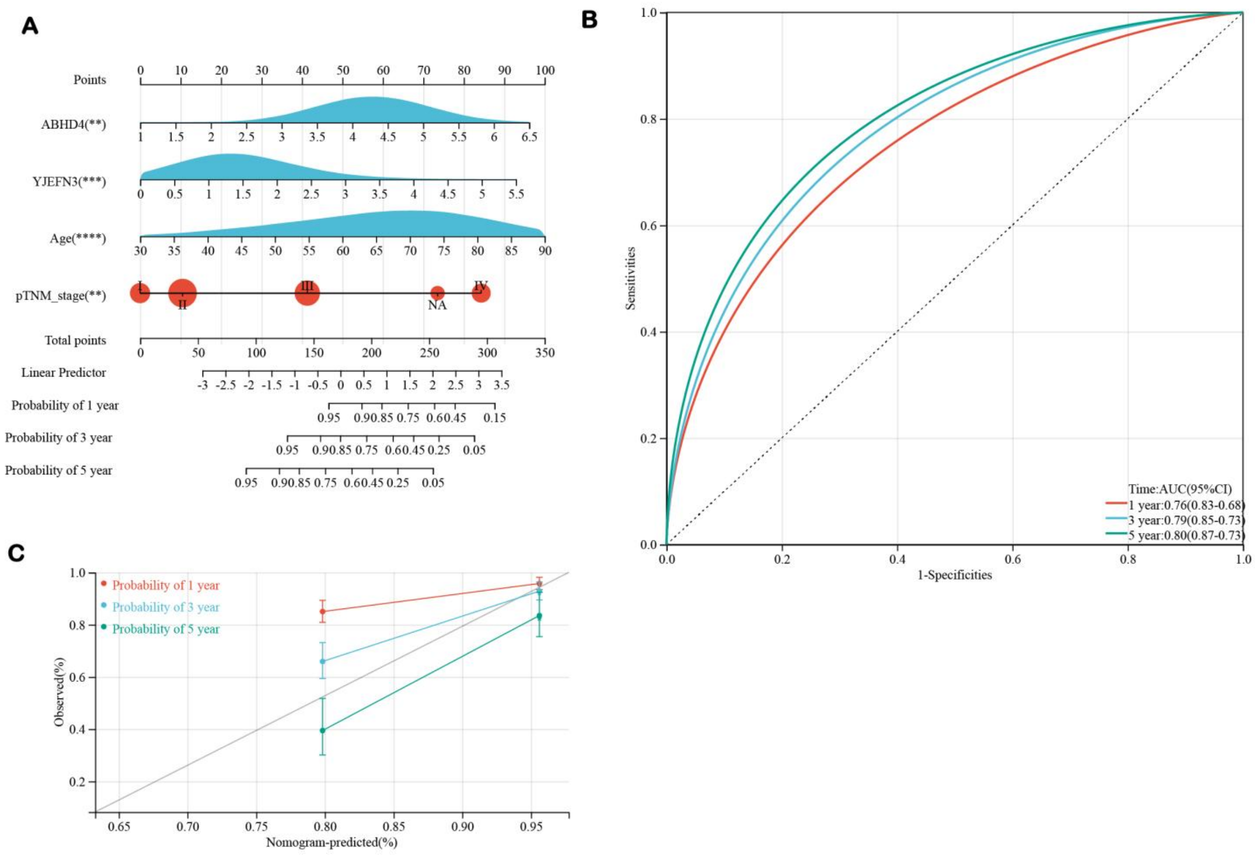

Figure S8. A prognostic nomogram was constructed by combining independently significant genes with essential clinical parameters to provide a quantitative tool for predicting patient survival.

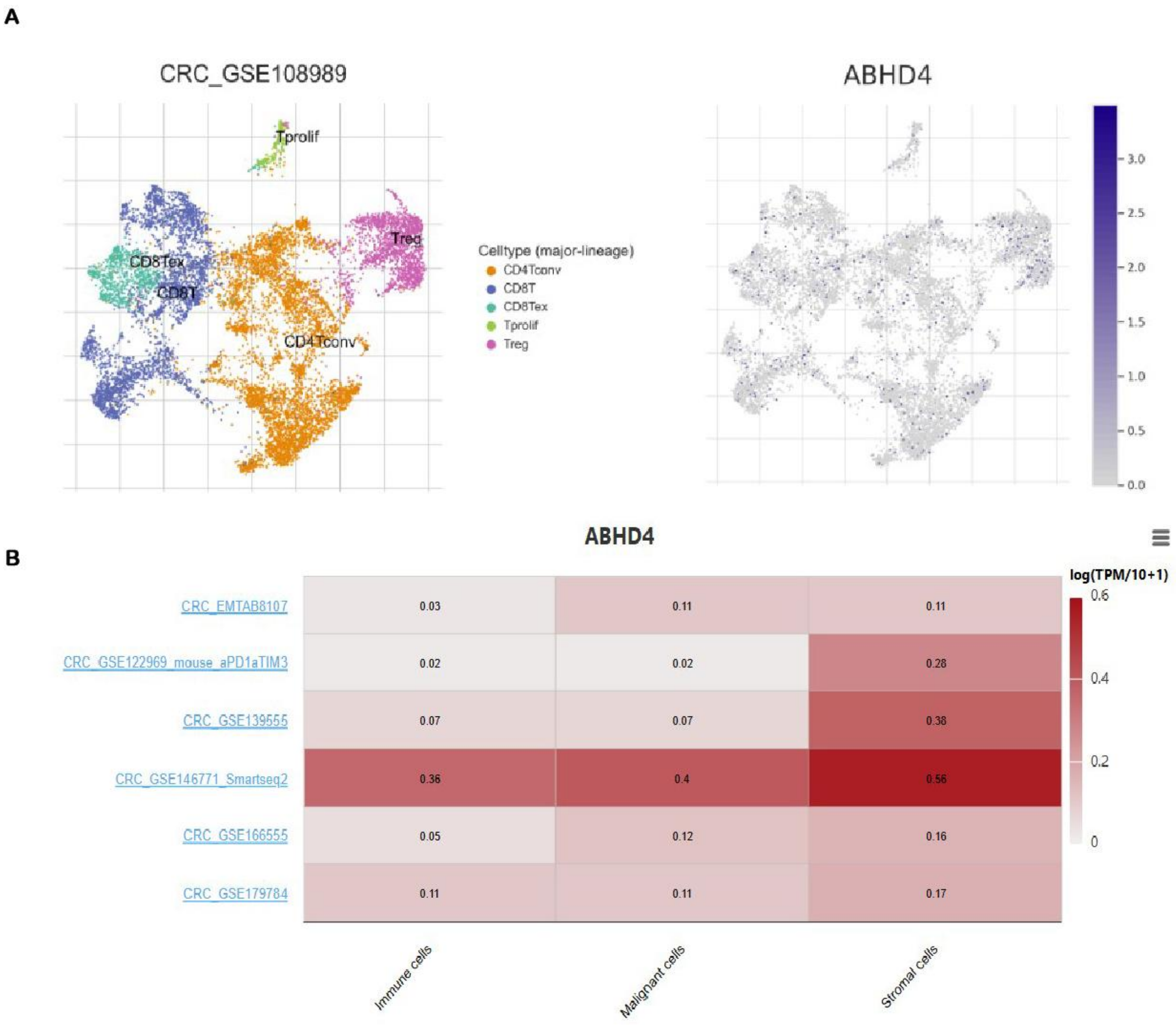

Figure S9. Analysis of independent prognostic biomarkers in the tumor microenvironment using the TISCH2 single-cell database.(A) The expression pattern of ABHD4 across all cell types in various colorectal cancer single-cell datasets.**(B)**Single-cell sequencing analysis of the expression of the ABHD4 gene across different cell types.

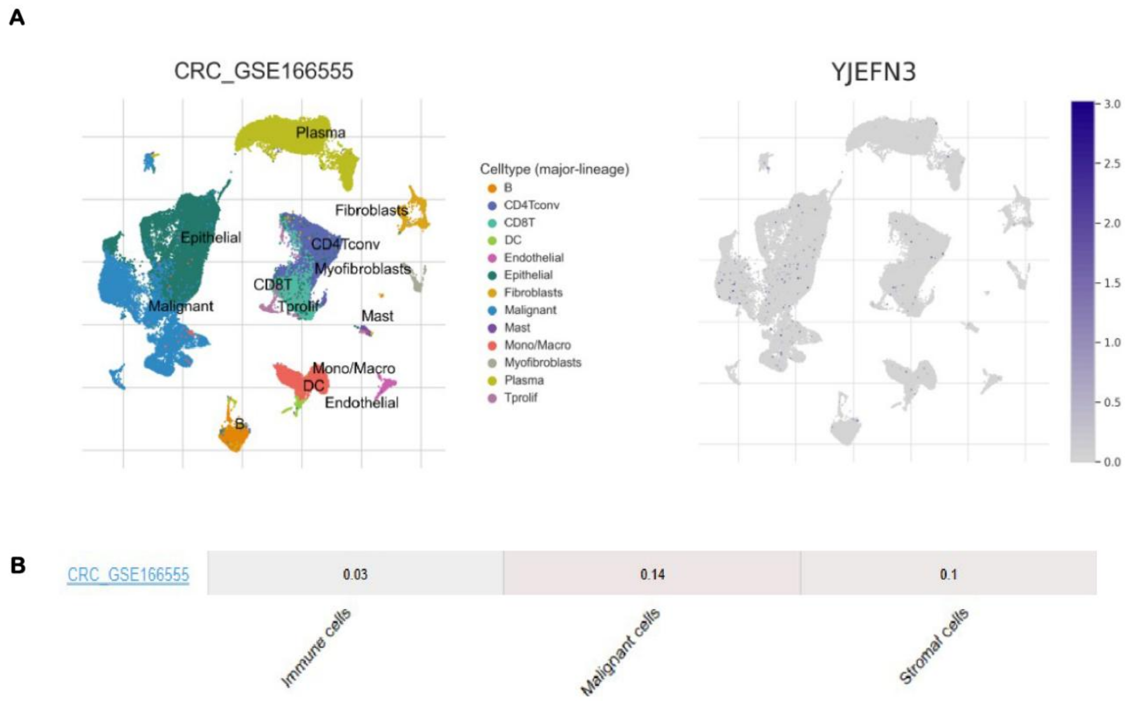

Figure S10. Analysis of independent prognostic biomarkers in the tumor microenvironment using the TISCH2 single-cell database.(A) The expression pattern of YJEFN3 across all cell types in various colorectal cancer single-cell datasets.**(B)**Single-cell sequencing analysis of expression of the YJEFN3 gene across different cell types.

Drug name	P_value	Q_value
Staurosporine_1034	1.2×10^{-3}	0.013386
Wee1 Inhibitor_1046	8.9×10^{-5}	0.026729
Nutlin-3a (-)_1047	3.5×10^{-3}	0.001591
Pictilisib_1058	2.0×10^{-5}	0.000320
5-Fluorouracil_1073	0.03	0.042979
BI-2536_1086	5.7×10^{-6}	0.000300
Oxaliplatin_1089	0.02	0.039158
Erlotinib_1168	5.3×10^{-7}	1.68×10^{-7}
AZ960_1250	1.4×10^{-4}	0.000409
AZD1332_1463	9.7×10^{-8}	6.13×10^{-5}
Pevonedistat_1529	4.0×10^{-3}	0.018392
Luminespib_1559	2.4×10^{-3}	0.030442
ERK_2440_1713	3.9×10^{-3}	0.012749
AZD5991_1720	1.6×10^{-4}	0.022126
TAF1_5496_1732	4.7×10^{-10}	0.000311
IGF1R_3801_1738	9.7×10^{-7}	0.000921
JAK_8517_1739	3.5×10^{-4}	0.006729
Dihydrorotenone_1827	2.6×10^{-10}	4.60×10^{-7}
OF-1_1853	2.0×10^{-9}	1.42×10^{-7}
BMS-754807_2171	1.7×10^{-10}	6.13×10^{-5}

Supplementary Table S1: p-values and FDR-corrected q-values corresponding to significant differences reported in the "Prediction of therapeutic sensitivity" section
